# Community pharmacists' knowledge, attitudes, and practices toward self-medication for common cold and influenza: A COM-B model–based cross-sectional study

**DOI:** 10.1016/j.rcsop.2026.100735

**Published:** 2026-04-02

**Authors:** Ema Pristi Yunita, Abdul Rahem, Novanto Yudistira, Riza Alfian, Suharjono

**Affiliations:** a Doctoral Program of Pharmaceutical Sciences, Faculty of Pharmacy, Universitas Airlangga, Surabaya, Indonesia; b Department of Pharmacy, Faculty of Medicine, Universitas Brawijaya, Malang, Indonesia; c Department of Pharmacy Practice, Faculty of Pharmacy, Universitas Airlangga, Surabaya, Indonesia; d Innovative Pharmacy Practice and Integrated Outcome Research (INACORE) Group, Universitas Airlangga, Surabaya, Indonesia; e Department of Informatics, Faculty of Computer Science, Universitas Brawijaya, Malang, Indonesia; f Department of Pharmacy, Sekolah Tinggi Ilmu Kesehatan ISFI Banjarmasin, Banjarmasin, Indonesia

**Keywords:** Pharmacists, Self-medication, COM-B model, Community pharmacy, Behavioral determinants

## Abstract

**Background:**

Community pharmacists play a crucial role in advising patients on self-medication for minor ailments such as the common cold and influenza. Howeve, behavioral factors influencing their consultation practices remain underexplored in Indonesia.

**Objective(s):**

To evaluate community pharmacists' knowledge, attitudes, and practices (KAP) regarding self-medication consultations for common cold and influenza, and to interpret these behaviors using the COM-B (Capability, Opportunity, Motivation–Behavior) framework.

**Methods:**

A cross-sectional survey was conducted among 221 community pharmacists in the Malang Region, East Java, Indonesia. A validated 67-item questionnaire assessed pharmacists' KAP. Data were analyzed using descriptive statistics, the item difficulty index (Pd), and the Relative Importance Index (RII), and multiple linear regression to identify behavioral predictors.

**Results:**

More than half of pharmacists demonstrated moderate knowledge levels (50.7%). Most knowledge items were moderately complex (Pd = 0.30–0.79). Pharmacists showed highly positive attitudes (RII > 0.90, range: 0.84–0.95) and generally good, though inconsistent, practice behaviors (median RII = 0.93, range: 0.50–0.95). Attitude was the strongest predictor of practice (β = 2.314, *p* < 0.001), whereas knowledge was not.

**Conclusion:**

Interpreted through the COM-B framework, Indonesian community pharmacists demonstrated moderate knowledge, positive attitudes, and generally good but inconsistent practices in self-medication consultations for the common cold and influenza. Attitude emerged as the primary behavioral determinant influencing practice. Interventions that enhance motivation, establish structured consultation guidelines, and integrate digital decision-support tools may strengthen responsible self-medication services in Indonesia.

## Highlights

• Pharmacists show moderate knowledge but highly positive attitudes toward self-medication.
• Motivation and opportunity factors jointly influence consultation behavior patterns.
• The COM-B model offers a behavioral lens for interpreting KAP survey findings.
• This study offers evidence from an LMIC context to inform behavior-based interventions.
• Digital decision-support and AI tools are proposed to enhance the quality of consultations.

## 1 Introduction

Common cold and influenza remain among the most common respiratory viral infections worldwide and are major contributors to morbidity, productivity loss, and inappropriate medicine use.^1^, ^2^, 3. In many low- and middle-income countries, including Indonesia, these illnesses are frequently self-managed using over-the-counter (OTC) products due to convenience, accessibility, and perceptions of mild disease severity.^4^^,^^5^ However, self-medication without adequate guidance may lead to incorrect drug selection, drug-drug interactions, misuse of combination products, and delays in seeking medical attention for more serious symptoms.^6^ Evidence from Indonesia and other low-resource settings shows that a poor understanding of influenza-like illness can lead to inappropriate self-medication behaviors, including the misuse of multi-ingredient products and antibiotics.^7^^,^^8^

Community pharmacists contribute significantly to responsible self-medication by assessing symptoms, selecting suitable non-prescription medicines, providing structured counseling, and referring clients when self-care is inappropriate.^9^^,^^10^ Evidence from Indonesia and comparable settings shows that pharmacist-led support enhances the appropriateness of treatment and promotes safe medicine use in the management of minor ailments.^11^^,^^12^ These responsibilities are particularly relevant in regional urban areas such as the Malang Region in East Java, Indonesia, where pharmacists frequently serve as the first point of contact for individuals seeking advice for respiratory symptoms. Despite this essential role, variations in practice quality persist. Studies have documented gaps in pharmacists' information-gathering, patient communication, and adherence to professional standards during minor ailment consultations.^13^ These challenges highlight the need to examine the behavioral factors that influence pharmacists' performance in routine practice.

Knowledge, attitudes, and practices (KAP) surveys are widely used to assess the preparedness of healthcare professionals, including community pharmacists, particularly in contexts requiring patient-facing communication and clinical judgment.^14^ Although useful, KAP studies often lack the explanatory depth required to understand why certain behaviors occur. The capability, opportunity, motivation–behavior (COM-B) model provides a complementary behavioral framework that identifies factors that enable or constrain individuals' actions.^15^ Recent applications of COM-B have demonstrated its relevance for understanding pharmacists' behavioral determinants, organizational influences, and readiness to deliver patient-centered services.^16^

Although substantial research has examined pharmacists' roles in minor ailment management, no published study in Southeast Asia has specifically assessed community pharmacists' knowledge, attitudes, and practices regarding self-medication for the common cold and influenza while integrating the COM-B behavioral model. Indonesian studies have primarily focused on antibiotics, cough management, or general self-medication patterns,^5^ leaving a limited understanding of pharmacists' decision-making processes for common respiratory viral infections. Global pharmacy frameworks have emphasized the need for stronger behavioral competencies and structured self-care support within community pharmacy practice, especially for conditions frequently mismanaged with OTC products.^17^^,^^18^

The novelty of this study lies in its integrated methodological approach, combining a traditional KAP framework with the COM-B behavioral model to examine pharmacists' self-medication consultation practices for the common cold and influenza. This dual-framework design moves beyond descriptive assessment and provides behavioral explanations of capability, opportunity, and motivation. To the best of current knowledge, this study is the first in Indonesia and the broader Southeast Asian region to provide a multidimensional behavioral analysis of pharmacists' support for self-medication for common viral respiratory infections.

Accordingly, this study aimed to evaluate community pharmacists' knowledge, attitudes, and practices regarding self-medication for the common cold and influenza, and to analyze behavioral determinants influencing their practice using the COM-B model. Findings are expected to inform the design of targeted educational initiatives, behavioral interventions, and policy strategies to strengthen responsible self-medication services in community pharmacy settings.

## 2 Methods

### 2.1 Study design and methods

A quantitative cross-sectional survey was conducted using a self-administered questionnaire to assess community pharmacists' knowledge, attitudes, and practices (KAP) related to self-medication consultations for the common cold and influenza. The study involved licensed community pharmacists working in retail pharmacies across the Malang Region, East Java, Indonesia, which includes Malang City (urban) and Malang Regency (suburban and rural). Data were collected between September and October 2025. The study followed the Strengthening the Reporting of Observational Studies in Epidemiology (STROBE) guidelines for design, implementation, and reporting (supplementary file 1).^19^

### 2.2 Sampling and recruitment of subjects

Community pharmacists practicing in the Malang Region formed the target population. Based on data from the Indonesian Pharmacists Association (IAI) Malang branch, 473 licensed pharmacists were identified. The minimum required sample was 217 pharmacists, calculated using the Slovin formula with a 5% margin of error.^20^ A convenience sampling approach was used to facilitate efficient recruitment, and the online questionnaire was distributed via professional WhatsApp groups coordinated by IAI Malang. When the initial response rate fell short of the target, snowball sampling was used, with participating pharmacists sharing the survey link with colleagues to broaden recruitment.

Inclusion criteria were pharmacists who provided pharmaceutical services in community pharmacies in Malang City or Malang Regency, had experience delivering self-medication consultations, completed the questionnaire in full, and provided informed consent. Incomplete submissions were excluded from the analysis.

### 2.3 Theoretical framework

This study applied the capability, opportunity, motivation–behavior (COM-B) model to interpret the behavioral determinants of pharmacists' self-medication practices. The COM-B model conceptualizes behavior as the interaction among capability, opportunity, and motivation, which together influence performance in real-world settings. The KAP framework was integrated with COM-B to enable theory-driven interpretation of survey findings. In this mapping, knowledge represented psychological capability, attitudes reflected reflective and automatic motivation, and practice corresponded to physical capability and social opportunity. This framework supported the identification of behavioral determinants influencing pharmacists' self-medication practices.

### 2.4 Instrument development

The KAP questionnaire was developed through a four-stage process to ensure validity and reliability. Item generation was conducted collaboratively by the principal researcher and two pharmacy academics, drawing on previous literature and self-medication consultation guidelines. Content validity was assessed by two independent experts, who evaluated each item for relevance, clarity, and representativeness. Face validity testing was performed with 30 pharmacists outside the Malang Region to confirm item comprehensibility and feasibility. The same group participated in reliability testing, with Cronbach's Alpha values above the accepted threshold (α > 0.60).

All items met the required criterion for item validity, with corrected item–total correlations exceeding *r* = 0.374 (*p* < 0.05; df = 28). For the knowledge domain (23 items), item–total correlations ranged from 0.403 to 0.761, and Cronbach's Alpha was 0.895. For the attitude domain (8 items), correlations ranged from 0.527 to 0.845, and Cronbach's Alpha was 0.850. For the practice domain (36 items), correlations ranged from 0.391 to 0.863, and Cronbach's Alpha was 0.949. These results confirm that the questionnaire was valid and reliable for use in the primary survey.

### 2.5 Data collection and analysis

Completed responses were exported from Google Forms into Microsoft Excel and analyzed using IBM SPSS Statistics version 27. Data were screened for completeness before analysis. Descriptive statistics summarized pharmacists' demographic and professional characteristics and generated mean scores for each KAP domain. Knowledge items (23 multiple-choice questions) were scored as 1 (correct) or 0 (incorrect), while attitude items (8 statements) and practice items (36 statements) were rated using four-point Likert scales (1 = strongly disagree to 4 = strongly agree).

Bloom's cut-off criteria were applied to categorize pharmacists' knowledge scores into three levels: good (≥ 80%), moderate (60–79%), and poor (< 60%). This classification approach has been widely adopted in KAP studies to facilitate the interpretation of knowledge scores and to identify potential gaps in respondents' understanding of specific health topics.^21^ Applying this classification enables researchers to interpret knowledge levels more systematically and to identify areas where targeted educational or practice-related interventions may be needed.

The complete KAP questionnaire comprised 67 items, all of which were completed before submission. Negatively worded practice items (items 16, 17, 21, 24, 27, 29, and 31) were reverse-coded so that “strongly disagree” received a score of 4 and “strongly agree” received a score of 1. Domain scores were then calculated by summing item responses.

For the knowledge domain, Pearson's chi-square or Fisher's exact test (when assumptions were unmet) compared correct and incorrect response distributions. The item difficulty index (Pd) was calculated as the proportion of correct answers, categorizing items as easy, moderate, or difficult.

For attitude and practice items, Kruskal–Wallis tests were used to examine differences across Likert-scale categories. Relative importance index (RII) values were computed to rank the perceived importance of each item (range: 0–1; higher scores indicate a greater extent). RII scores were interpreted contextually; lower RII values, which did not necessarily indicate suboptimal practice, were examined in relation to clinical appropriateness and rational professional behavior.

Multiple linear regression was performed to identify predictors of pharmacists' knowledge, attitude, and practice scores, with sociodemographic and inter-domain KAP variables entered as independent variables. Regression coefficients (β), standard errors (SE), and *p*-values were reported, with statistical significance set at *p* < 0.05. Nonparametric tests (Mann–Whitney U or Kruskal–Wallis) were used to compare KAP scores across demographic subgroups and professional characteristics, given nonnormal distributions. This analytical approach provided both descriptive and inferential insights, enabling the identification of behavioral determinants within the COM-B framework.

### 2.6 Ethical approval

Ethical approval was obtained from the Health Research Ethics Committee, Faculty of Medicine, Universitas Brawijaya, Malang, Indonesia (Approval No. 100/EC/KEPK–S3/05/2025). The study adhered to the principles of the Declaration of Helsinki. All participants received an online information sheet describing the study objectives, confidentiality procedures, and their right to withdraw at any time. Completion of the questionnaire was regarded as informed consent. No personal identifiers were collected, and all data were stored securely and analyzed anonymously to ensure confidentiality.

## 3 Results

A total of 221 community pharmacists participated in the survey, exceeding the minimum required sample size of 217, which was calculated using the Slovin formula based on the regional pharmacist population (*N* = 473). The high response rate strengthened the sample's representativeness across both urban and rural practice settings.

### 3.1 Participant characteristics

As shown in Table 1, the majority of participants were identified as female (81.9%). The mean (±SD) age was calculated as 33.5 ± 7.9 years, with most pharmacists categorized in the 23–33-year age group (64.7%), followed by the 34–44-year age group (27.6%). More than half were based in Malang Regency (52.0%), while 48.0% were employed in Malang City. A bachelor's degree in pharmacy was held by 89.1% of participants, 10.4% were recorded as having a master's degree, and 0.5% were found to hold a doctoral degree. A large proportion were classified as pharmacists-in-charge (86.9%), and 72.9% reported working in pharmacies they personally owned. In terms of practice setting, 76.0% were employed in independent pharmacies, while 24.0% were working in banner pharmacies. Regarding professional experience, 43.0% of pharmacists reported practicing for 1–5 years, 23.5% for 6–10 years, and 14.0% for more than 10 years. Approximately 67.9% reported practicing ≥5 h per day, with 34.8% engaging in 25–35 h of weekly work.

**Table 1:** Characteristics of participants.

Characteristic	*n* = 221 (%)
Gender	
Female	181 (81.9)
Male	40 (18.1)
Age, years old	
Mean ± SD	33.5 ± 7.9
Minimum / Maximum	23 / 76
23–33	143 (64.7)
34–44	61 (27.6)
45–55	11 (5.0)
56–66	5 (2.3)
67–77	1 (0.4)
Pharmacy location	
Malang Regency	115 (52.0)
Malang City	106 (48.0)
Educational level	
Licensed pharmacist	197 (89.1)
Licensed pharmacist with a master's degree	23 (10.4)
Licensed pharmacist with a doctoral degree	1 (0.5)
⁎Pharmacist position	
Pharmacist-in-charge	192 (86.9)
Supporting pharmacist	29 (13.1)
Ownership status of the pharmacy	
Owned by the pharmacist practising in the pharmacy	161 (72.9)
Owned by another individual	60 (27.1)
Pharmacy type	
Independent pharmacy	168 (76.0)
Banner pharmacy	53 (24.0)
Professional experience in community pharmacy practice	
1–6 months	23 (10.4)
7–11 months	20 (9.0)
1–5 years	95 (43.0)
6–10 years	52 (23.5)
More than 10 years	31 (14.0)
Average duration of daily practice	
≥ 5 h	150 (67.9)
< 5 h	71 (32.1)
Average weekly working hours in community pharmacy	
< 25 h	53 (24.0)
25–35 h	77 (34.8)
36–40 h	35 (15.8)
> 40 h	56 (25.3)

⁎ Note: In Indonesia, the pharmacist-in-charge *(Apoteker Penanggung Jawab Apotek)* is the pharmacist legally responsible for managing a pharmacy and ensuring regulatory compliance, while the supporting pharmacist *(Apoteker Pendamping)* is a licensed practitioner who assists in service delivery under their coordination, as outlined in national regulations.

### 3.2 Knowledge of common cold and influenza management (capability)

Based on item-level analysis in Table 2, most knowledge questions were categorized as moderate difficulty (Pd = 0.30–0.79). Only a few questions were identified as easy (Pd ≥ 0.80), including identifying the pathogens causing the common cold (Pd = 0.89; *p* = 0.371), influenza (Pd = 0.95; *p* = 0.228), and recognizing Reye's syndrome–related risks (Pd = 0.90; *p* < 0.001) and ibuprofen side effects (Pd = 0.86; *p* = 0.033). In contrast, some items were classified as difficult (Pd < 0.30), such as identifying influenza symptoms (Pd = 0.21; *p* = 0.854) and determining safe medications for breastfeeding women (Pd = 0.25; *p* = 0.718). Moderate-difficulty items were represented by questions on zinc supplementation (Pd = 0.43; *p* = 0.037), safety of cold medications in hypertension (Pd = 0.76; *p* = 0.011), management of pregnant women (Pd = 0.67; *p* = 0.196), and the selection of oral decongestants for patients with comorbidities including chronic bronchitis, hyperthyroidism, lung cancer, or chronic kidney disease (Pd = 0.38; *p* = 0.250). Based on Bloom's cut-off criteria, pharmacists' overall knowledge was categorized as moderate in 50.7% of respondents (Appendix 3), followed by good (26.7%) and poor (22.6%).

**Table 2:** Knowledge of community pharmacists regarding the common cold and influenza syndrome.

Questions	True n (%)	False n (%)	Pd	*p-value*
What is the pathogen that causes the common cold syndrome?	196 (88.7)	25 (11.3)	0.89	0.371^a^
What is the pathogen that causes the influenza syndrome?	209 (94.6)	12 (5.4)	0.95	0.228^a^
What are the symptoms of the common cold syndrome?	132 (59.7)	89 (40.3)	0.60	0.703^b^
What are the symptoms of the influenza syndrome?	46 (20.8)	175 (79.2)	0.21	0.854^b^
Which cold medication is safe for pregnant women?	148 (67.0)	73 (33.0)	0.67	0.196^b^
Which cold medication is safe for breastfeeding women?	55 (24.9)	166 (75.1)	0.25	0.718^b^
What medication is used to relieve muscle pain caused by influenza?	212 (95.9)	9 (4.1)	0.96	0.828^a^
What medication is used to relieve muscle pain due to influenza in patients with a history of asthma?	161 (72.9)	60 (27.1)	0.73	0.313^b^
What medication is used to treat cough in patients who require a mucolytic?	189 (85.5)	32 (14.5)	0.86	0.161^a^
What medication is used to relieve nasal congestion in patients with uncontrolled hypertension?	167 (75.6)	54 (24.4)	0.76	**0.011**^b^*
Which of the following comorbid conditions, namely chronic bronchitis, hyperthyroidism, lung cancer, or chronic kidney disease, should be considered by pharmacists when selecting oral decongestants for patients with common cold syndrome?	84 (38.0)	137 (62.0)	0.38	0.250^b^
What is the benefit of administering zinc supplements to patients with the common cold and/or influenza syndrome?	95 (43.0)	126 (57.0)	0.43	**0.037**^b^*
What is the class of drugs that work by breaking the protein bonds in mucus to facilitate sputum expulsion?	185 (83.7)	36 (16.3)	0.84	0.256^b^
What is the class of drugs that act by stimulating alpha-1 adrenergic receptors?	192 (86.9)	29 (13.1)	0.87	**0.019**^a^*
What is the mechanism of action of decongestant drugs?	159 (71.9)	62 (28.1)	0.72	0.538^b^
What is the recommended daily dose (mg) of vitamin C after the onset of symptoms to reduce the severity and duration of common cold and/or influenza symptoms?	143 (64.7)	78 (35.3)	0.65	0.651^b^
What is the maximum daily dose (g) of paracetamol for fever in adults without a history of alcoholism?	173 (78.3)	48 (21.7)	0.78	**0.003**^b^*
What is the maximum daily dose (g) of paracetamol for fever in adults with a history of alcoholism?	154 (69.7)	67 (30.3)	0.70	0.741^b^
What is the maximum daily dose (g) of paracetamol for fever in children aged 8–10 years?	119 (53.8)	102 (46.2)	0.54	0.921^b^
Which medication is contraindicated for an 8-year-old child with a fever due to influenza?	146 (66.1)	75 (33.9)	0.66	**0.004**^b^*
Which medication may cause Reye's syndrome if given to a child with influenza?	199 (90.0)	22 (10.0)	0.90	**< 0.001**^a^*
Reye's syndrome is a serious condition that can cause organ damage. Which organ is affected?	193 (87.3)	28 (12.7)	0.87	0.226^a^
What short-term side effect may occur after administering ibuprofen to relieve fever in adults or children?	191 (86.4)	30 (13.6)	0.86	**0.033**^a^*

Pd = item difficulty index, Pd 0.80–1.00 indicated easy questions, Pd 0.30–0.79 indicated moderate questions, and Pd < 0.30 indicated difficult questions.

a Fisher's exact test was applied, categorized according to duration of professional experience. * *p*-value <0.05 was considered statistically significant.

b Pearson's chi-square test was applied, categorized according to duration of professional experience. * p-value <0.05 was considered statistically significant.

### 3.3 Attitude toward self-medication consultation (motivation)

Pharmacists generally showed positive attitudes toward self-medication services, as presented in Table 3. The highest-ranked statement by RII (0.95) indicated that pharmacists ensured the accuracy of information provided to patients. High agreement levels were also reported for providing medication information politely (RII = 0.94) and delivering services rationally (RII = 0.93). Pharmacists also agreed with the usefulness of consultation support tools such as digital health applications and printed brochures (RII = 0.87), although this item ranked lower than other attitude statements. The lowest-ranked statement concerned participation in professional development activities (RII = 0.84). Most respondents strongly agreed that pharmacists should guide patients appropriately and consult the latest references when providing advice (RII range: 0.84–0.95). No significant differences in attitude scores were observed across demographic variables (*p* > 0.05; Appendix 1).

**Table 3:** Attitude of community pharmacists regarding the common cold and influenza syndrome.

Statements	Strongly disagree n (%)	Disagree n (%)	Agree n (%)	Strongly agree n (%)	RII	SD	Rank	*p-value*
I used the latest and most relevant references as guidance when providing information about medications for the common cold and/or influenza.	2 (0.9)	6 (2.7)	70 (31.7)	143 (64.7)	0.90	0.59	6	0.065
I ensured the accuracy of the information provided to patients regarding the use of medications for the common cold and/or influenza.	2 (0.9)	1 (0.5)	32 (14.5)	186 (84.2)	0.95	0.46	1	0.488
I needed self-medication consultation support tools (such as brochures or health applications) to provide information about common cold and/or influenza medications to patients.	1 (0.5)	11 (5.0)	94 (42.5)	115 (52.0)	0.87	0.61	7	0.163
I actively participated in seminars, webinars, and interprofessional discussions to increase my knowledge about managing common cold and/or influenza syndromes.	2 (0.9)	20 (9.0)	96 (43.4)	103 (46.6)	0.84	0.68	8	0.541
I demonstrated confidence when providing self-medication consultation services to patients regarding to the management of common colds and/or influenza.	1 (0.5)	3 (1.4)	72 (32.6)	145 (65.6)	0.91	0.54	4	0.528
I provided self-medication services for the common cold and/or influenza in a rational manner.	2 (0.9)	1 (0.5)	50 (22.6)	168 (76.0)	0.93	0.51	3	0.269
I always explained the directions for using medications for the common cold and/or influenza to patients in a clear and concise manner.	1 (0.5)	5 (2.3)	64 (29.0)	151 (68.3)	0.91	0.55	5	0.548
I always provided patients with information on how to use medications for common colds and/or influenza politely and respectfully.	1 (0.5)	1 (0.5)	47 (21.3)	172 (77.8)	0.94	0.47	2	0.651

The Kruskal–Wallis test was applied, with RII representing the relative importance index and SD representing the standard deviation, categorized according to the duration of professional experience. * p-value <0.05 was considered statistically significant.

### 3.4 Practice of self-medication consultation (behavioral output)

As summarized in Table 4 and detailed in Appendix 2, pharmacists generally demonstrated patient-centered practices in self-medication consultations. The highest-ranked items included asking about patient symptoms (RII = 0.95), checking dosages before dispensing (RII = 0.94), and providing instructions for ibuprofen use (RII = 0.94). Some misconceptions were identified, including the advice that paracetamol should be taken after meals (RII = 0.78). The lowest-ranked practices were dispensing antibiotics for sore throat (RII = 0.50), recommending N95 mask use (RII = 0.65; *p* = 0.048), and asking adult patients about any history of alcoholism (RII = 0.78). A significant difference was observed only for the recommendation to wear N95 masks (p = 0.048) when categorized by years of professional experience.

**Table 4:** Practices of community pharmacists regarding the common cold and influenza syndrome.

Statements	Strongly disagree n (%)	Disagree n (%)	Agree n (%)	Strongly agree n (%)	RII	SD	Rank	*p-value*
I conducted patient assessments (gathering information related to the patient's condition) to determine the appropriate medication for the common cold and/or influenza.	1 (0.5)	2 (0.9)	74 (33.5)	144 (65.2)	0.91	0.53	9	0.435
I asked patients about the symptoms they experienced.	1 (0.5)	1 (0.5)	42 (19.0)	177 (80.1)	0.95	0.45	1	0.618
I asked patients about the duration of their symptoms.	1 (0.5)	1 (0.5)	55 (24.9)	164 (74.2)	0.93	0.48	5	0.350
I asked patients about their age.	1 (0.5)	1 (0.5)	65 (29.4)	154 (69.7)	0.92	0.50	7	0.432
I asked female adult patients whether they were pregnant or breastfeeding.	1 (0.5)	2 (0.9)	50 (22.6)	168 (76.0)	0.94	0.49	4	0.870
I asked patients whether they were currently using or had recently used medications for the common cold and/or influenza.	1 (0.5)	3 (1.4)	59 (26.7)	158 (71.5)	0.92	0.52	8	0.087
I checked the accuracy of the dosage and administration of medications for the common cold and/or influenza before dispensing them to children.	1 (0.5)	1 (0.5)	51 (23.1)	168 (76.0)	0.94	0.48	2	0.912
I informed patients that ibuprofen should be taken after meals.	1 (0.5)	1 (0.5)	51 (23.1)	168 (76.0)	0.94	0.48	2	0.363
I advised patients to get adequate rest and sleep during the common cold and/or influenza.	0 (0.0)	3 (1.4)	56 (25.3)	162 (73.3)	0.93	0.48	5	0.216
I advised patients to avoid respiratory irritants, such as smoke, air pollution, and dust, during the common cold and/or influenza.	0 (0.0)	7 (3.2)	67 (30.3)	147 (66.5)	0.91	0.54	10	0.119

The Kruskal–Wallis test was applied, with RII representing the relative importance index and SD representing the standard deviation, categorized according to the duration of professional experience. * p-value <0.05 was considered statistically significant.

### 3.5 Predictors of KAP scores

The multivariate linear regression analysis (Table 5) showed that in the knowledge domain, age (β = −0.125; *p* < 0.001) and pharmacy location (β = −1.184; *p* = 0.014) were significant negative predictors. For the attitude domain, practice was the only significant predictor (β = 0.236; *p* < 0.001), while in the practice domain, attitude was the sole predictor (β = 2.314; p < 0.001). All regression models were statistically significant (Knowledge: R^2^ = 0.220, *p* < 0.001; Attitude: R^2^ = 0.572, p < 0.001; Practice: R^2^ = 0.572, p < 0.001).

**Table 5:** Predictors of pharmacists' KAP scores.

Predictor variables	Knowledge			Attitude			Practice		
	β	SE	*p-value*	β	SE	*p-value*	β	SE	*p-value*
KAP-related predictors									
Knowledge	–			0.050	0.044	0.256	0.105	0.138	0.448
Attitude	0.125	0.109	0.256	–			2.314	0.147	**<0.001**⁎
Practice	0.027	0.035	0.448	0.236	0.015	**<0.001**⁎	–		
Sociodemographic factors									
Age	−0.125	0.036	**<0.001**⁎	0.002	0.024	0.935	0.110	0.074	0.137
Gender	−0.451	0.598	0.451	0.095	0.379	0.802	0.441	1.188	0.711
Educational and professional background									
Educational level	1.086	0.762	0.155	−0.457	0.484	0.346	0.863	1.519	0.571
Professional experience (duration of practice)	−0.443	0.252	0.081	0.087	0.161	0.588	−0.637	0.503	0.207
Pharmacist position	1.167	0.726	0.109	0.613	0.461	0.185	−1.554	1.447	0.284
Workplace and organizational characteristics									
Pharmacy location	−1.184	0.476	**0.014**⁎	0.010	0.306	0.974	0.143	0.960	0.882
Pharmacy type	−1.171	0.618	0.060	0.256	0.395	0.517	−0.059	1.238	0.962
Ownership status	0.692	0.585	0.238	0.131	0.372	0.725	0.085	1.166	0.942
Average duration of daily practice	0.578	0.590	0.328	−0.205	0.375	0.584	0.489	1.174	0.678
Average weekly working hours	0.066	0.263	0.803	−0.048	0.167	0.773	0.403	0.522	0.441

Multiple linear regression was applied separately for each dependent variable (Knowledge, Attitude, Practice).

Model fit statistics: Knowledge model: R^2^ = 0.220, Adjusted R^2^ = 0.175, F(12, 207) = 4.873, p < 0.001; Attitude model: R^2^ = 0.572, Adjusted R^2^ = 0.547, F(12, 207) = 23.058, *p* < 0.001; Practice model: R^2^ = 0.572, Adjusted R^2^ = 0.547, F(12, 207) = 23.069, *p* < 0.001.

⁎ p < 0.05 was considered statistically significant.

### 3.6 Comparative analysis across demographic and professional groups

Comparative analyses (Appendix 1) showed significant differences in knowledge scores by age (p < 0.001), pharmacist position (*p* = 0.004), ownership status (*p* = 0.022), and professional experience (p < 0.001). Younger pharmacists (23–33 years) and those with shorter professional experience recorded higher median knowledge scores (p < 0.001). Supporting pharmacists and those working in non-owner-managed pharmacies also showed higher knowledge scores. No statistically significant differences were observed for attitude or practice scores across demographic and professional variables (*p* > 0.05).

### 3.7 Mapping findings to the COM-B framework

**Fig. 1:**
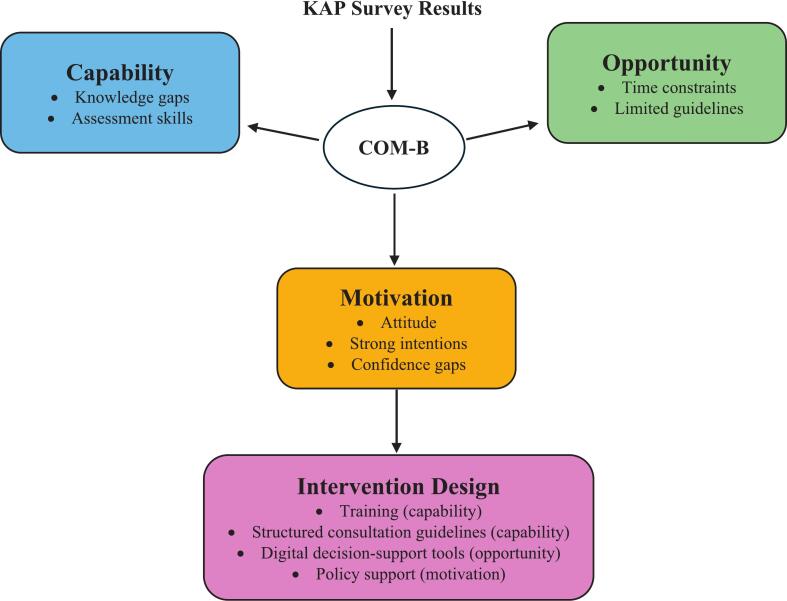
Mapping KAP survey to the COM-B framework and interventions.

Collectively, pharmacists demonstrated strong motivation and moderate capability, but opportunity-related constraints limited the consistent application of knowledge in practice. Behavioral performance, as illustrated in Fig. 1, reflected the interaction of personal and contextual factors. Although motivation and psychological capability were present, external barriers such as time pressure, limited staffing, and the absence of digital support tools restricted optimal practice.

## 4 Discussion

### 4.1 Overview of the main findings

This study found that community pharmacists had moderate knowledge, highly positive attitudes, and generally good but inconsistent practices in providing self-medication consultations for the common cold and influenza. Knowledge gaps were most evident in distinguishing symptoms and advising special populations. These patterns are consistent with previous reports highlighting pharmacists' difficulties in applying clinical knowledge to patient-specific decision-making.^11^^,^^22^ Attitudes were strongly positive, with openness to using digital tools and printed materials to support communication, aligning with earlier findings on pharmacists' receptiveness to technology-enabled interactions in community practice.^23^ Practice behaviors demonstrated active patient assessment but remained inconsistent due to opportunity-related constraints, including limited time, patient expectations, and the absence of standardized guidelines, similar to barriers reported in Jordan and Malaysia.^22^^,^^24^ Regression analysis further indicated that knowledge influenced attitudes, while attitudes predicted practice, reflecting the capability–motivation–behavior pathway described in the COM-B framework. Overall, pharmacists' responses reflected strong motivational commitment but also indicated the need for enhanced support systems to achieve consistent consultation performance.

In the Indonesian context, a middle-income country with rapidly expanding community pharmacy networks, these findings are particularly relevant. The Malang Region, an urban–rural transition area in East Java, reflects national patterns where limited resources, uneven access to continuing education, and varied local policies shape pharmacists' behavioral performance. Pharmacists in this region demonstrate strong foundational capabilities but require stronger support systems, including training, structured consultation guidelines, and digital decision-support tools. This pattern aligns with global evidence indicating that optimizing self-medication services requires strengthening both professional capabilities and external opportunities.^25^

### 4.2 KAP domains linked to COM-B elements

#### 4.2.1 Knowledge and the cognitive capability dimension

Pharmacists demonstrated moderate knowledge (50.7%), indicating adequate theoretical understanding of common cold and influenza management, but incomplete mastery of specific clinical areas. They performed well in identifying pathogens, contraindications, and adverse drug reactions, including issues related to Reye's syndrome and ibuprofen. They also showed moderate understanding of medication safety in special populations, such as patients with hypertension or other chronic conditions, reflecting foundational competence in clinical pharmacy. However, difficulties remained in distinguishing between the symptoms of the common cold and influenza, as well as in advising breastfeeding women. These gaps align with prior findings highlighting persistent challenges in pharmacists' applied clinical knowledge and patient assessment during self-medication consultations.^26^^,^^27^

Differences in knowledge scores were influenced by exposure to updated information and workplace dynamics. Younger, less experienced pharmacists achieved higher scores, likely because of their more recent academic training. Supporting pharmacists and those working in non-owner-managed pharmacies also recorded higher knowledge scores, possibly reflecting greater opportunities for peer interaction and shared professional decision-making within collaborative professional settings.^28^^,^^29^ Longer experience did not correspond with higher theoretical knowledge, highlighting the importance of ongoing education to keep pace with evolving recommendations.

From the COM-B perspective, these findings reflect limitations in psychological capability, as pharmacists' theoretical competence does not consistently translate into patient-specific decision-making. Strengthening cognitive capability through targeted training, updated clinical resources, and locally adapted guidelines is essential to support more accurate assessments and improve consultation quality in Indonesian community pharmacies.^25^ In addition, the findings suggest that targeted updates to evidence-based recommendations and pharmacist training resources may be needed to strengthen pharmacists' confidence and decision-making in upper respiratory infection (URI)-related self-care consultations.

#### 4.2.2 Attitude and the motivational dimension

Pharmacists demonstrated highly positive attitudes toward self-medication consultations, reflected in high RII scores and a strong sense of professional responsibility. They valued accuracy, politeness, and rational advice provision, which are consistent with reflective motivation in the COM-B model. Many respondents also expressed openness to using consultation support tools, including printed materials and health applications, to support communication with patients, consistent with earlier findings on community pharmacists' receptiveness to technology-assisted interactions.^23^ This receptiveness indicates intrinsic motivation and readiness for behavioral change when supportive tools are available. However, lower participation in professional development activities suggests that time pressures, workload, and limited access to training may weaken sustained motivation.^30^^,^^31^ Within Indonesia's community pharmacy environment, motivation therefore requires both intrinsic drivers, such as professional pride and commitment to patient safety, and extrinsic enablers, including accessible continuing education, mentorship, and supportive regulatory frameworks.

#### 4.2.3 Practice as the behavioral output influenced by opportunity

Pharmacists demonstrated strong behavioral performance, as reflected in active, patient-centered practices such as symptom assessment, dosage verification, and medication-use instruction. However, inconsistencies remained in several areas, including incomplete patient history-taking, occasional inaccuracies in medication administration advice (e.g., unnecessary instructions such as advising paracetamol to be taken after meals), and limited preventive counseling. These gaps correspond to opportunity-related constraints, particularly time pressure, patient expectations, and the absence of standardized consultation guidelines, similar to barriers reported in Jordan and Malaysia.^22^^,^^24^ The relatively low RII score for recommending N95 masks was not interpreted as inadequate counseling but rather as evidence of proportionate, evidence-based recommendations, since surgical masks are sufficient for preventing droplet transmission of common cold and influenza viruses.^6^

From the COM-B perspective, pharmacists demonstrated the capability and motivation to provide self-medication consultations. However, external opportunity constraints, such as limited consultation time, lack of structured practice protocols, and inconsistent access to updated resources, may hinder consistent performance. International evidence similarly highlights how workload, limited privacy, and insufficient access to evidence-based protocols constrain the delivery of high-quality self-care advice.^31^ Enhancing consultation guidelines, improving workflow support, and integrating digital decision-support systems may strengthen the efficiency and consistency of self-medication services in community pharmacies.

### 4.3 Integration of KAP findings within the COM-B framework

Integrating the KAP findings with the COM-B framework showed that attitude, representing motivation, was the only significant predictor of practice. At the same time, knowledge as an indicator of capability did not significantly influence either attitude or practice. This pattern suggests that cognitive understanding alone does not drive pharmacists' consultation behavior, supporting the COM-B principle that behavior emerges from the interaction of capability, motivation, and opportunity.^15^ Pharmacists with strong professional attitudes were more likely to demonstrate patient-centered behaviors, emphasizing accuracy, politeness, and rational medicine use. These findings align with behavioral research indicating that motivation and perceived control act as the proximal determinants of pharmacists' actions.^32^^,^^33^

Opportunity constraints, including limited time, staffing shortages, and a lack of structured consultation guidelines, may weaken the translation of capability and motivation into practice. Similar barriers have been reported internationally, including workload, limited privacy, and commercial pressures, which hinder consistent self-care consultations.^34^

In summary, integrating the KAP and COM-B frameworks provides a more complete understanding of pharmacists' behavioral mechanisms, and this integrative approach remains underexplored in Indonesia. Strengthening motivation through professional development and recognition, supported by improved opportunity structures, is essential for sustainable improvements in consultation practices.^25^

### 4.4 Implications for practice, education, and policy

These findings highlight practical, educational, and policy implications for strengthening pharmacists' self-medication consultation services in Indonesia. Although pharmacists demonstrated strong motivation and moderate capability, practice performance may still be influenced by contextual constraints such as time limitations, workload, and limited consultation resources.^12^^,^^35^ Addressing these challenges requires coordinated strategies targeting all components of the COM-B model. These findings indicate the need for targeted interventions to strengthen capability and opportunity, including training, structured consultation guidelines, digital decision-support tools, and policy support.

In practice, behavioral inconsistencies indicate the need for structured consultation guidelines to support consistent, patient-centered decision-making. Standardized counseling frameworks have been shown to improve clinical reasoning, patient safety, and service quality in community pharmacies.^36^^,^^37^ Integrating digital decision-support tools may help pharmacists manage informational and time constraints while improving service accuracy and efficiency.^38^^,^^39^ Emerging approaches increasingly incorporate artificial intelligence techniques, including expert systems and large language models, to support clinical decision-making in healthcare.^40^ Such innovations can further strengthen pharmacists' role as gatekeepers of responsible self-medication in Indonesia.^12^

Educationally, continuous professional development should emphasize communication skills, clinical assessment, and evidence-based decision-making, supported by behavioral frameworks such as COM-B and case-based learning approaches.^41^ At the policy level, integrating self-medication consultation into national pharmacy practice standards and providing institutional support may expand pharmacists' contributions to primary healthcare systems, particularly in low- and middle-income countries.^18^^,^^42^

### 4.5 Strengths and limitations

This study has several strengths that enhance its methodological rigor and contextual relevance. To the best of current knowledge, this is the first quantitative study in Indonesia to examine pharmacists' behavioral mechanisms in providing self-medication consultations for the common cold and influenza using an integrated KAP model and the COM-B framework. This theory-informed approach enabled a structured understanding of how capability, motivation, and opportunity interact to shape consultation behaviors, an approach increasingly recommended in pharmacy practice research.^33^^,^^43^

The relatively large sample size (*n* = 221) and inclusion of pharmacists from both urban and suburban community pharmacies in Malang improved the representativeness of the findings. The validated KAP instrument, supported by content validity indices and Cronbach's α, further ensured measurement reliability. Additionally, the study contributes evidence from a middle-income country context, where pharmacists often serve as accessible primary healthcare providers.^44^

However, the cross-sectional design limits causal inference, and the self-administered questionnaire may be subject to social desirability bias. Future research using longitudinal or mixed-method approaches could provide deeper behavioral insights.

### 4.6 Future directions

Future research will involve the design and evaluation of artificial intelligence–based tools, including an expert system and a large language model (LLM) integrated into a social robot platform, to assist pharmacists during self-medication consultations. Building on the current KAP findings, these AI-driven initiatives will support the development of behaviorally informed, technology-enabled interventions aimed at strengthening pharmacists' capability, motivation, and opportunity in real-world practice. In the long term, this research trajectory is expected to contribute to more efficient, interactive, and patient-centered models of community pharmacy care.

## 5 Conclusion

This study provides empirical evidence on Indonesian community pharmacists' knowledge, attitudes, and practices regarding self-medication consultations for the common cold and influenza, interpreted through the COM-B behavioral framework. Pharmacists demonstrated moderate knowledge, positive attitudes, and generally good practices, although variations in practice were observed. Attitude emerged as the strongest behavioral determinant influencing consultation behavior. These findings indicate that strengthening motivational factors and improving supportive practice environments are essential for enhancing the consistency of pharmacists' consultations. Interventions such as structured consultation guidelines, continuous professional development, and digital decision-support systems may help pharmacists manage information and time constraints while supporting more standardized, patient-centered self-medication services. Future research should evaluate how such behavioral and technological strategies can optimize pharmacists' contributions to responsible self-medication and primary healthcare delivery.

## Funding

This study was financially supported by the Indonesian Education Scholarship (Beasiswa Pendidikan Indonesia), the Center for Higher Education Funding and Assessment, 10.13039/501100002385Ministry of Higher Education, Science, and Technology, Republic of Indonesia, and the Indonesian Endowment Fund for Education, 10.13039/501100005045Ministry of Finance, Republic of Indonesia (Grant No. 00557/BPPT/BPI.06/01/2025). The funders had no role in the study design, data collection, analysis, interpretation, or manuscript preparation.

## Provenance and peer review

Not commissioned; externally peer reviewed.

## Patient consent for publication

Not required.

## CRediT authorship contribution statement

**Ema Pristi Yunita:** Writing – original draft, Visualization, Methodology, Investigation, Formal analysis, Data curation, Conceptualization. **Abdul Rahem:** Writing – review & editing, Validation, Supervision, Project administration, Methodology. **Novanto Yudistira:** Writing – review & editing, Supervision, Software, Methodology, Formal analysis. **Riza Alfian:** Writing – review & editing, Validation, Data curation. **Suharjono:** Writing – review & editing, Validation, Supervision, Funding acquisition, Conceptualization.

## Declaration of generative AI and AI-assisted technologies in the writing process

During the preparation of this work, the authors used ChatGPT (OpenAI, version GPT-5.3, 2026) to enhance the clarity and readability of the writing. After using this tool/service, the authors reviewed and edited the content as needed and take full responsibility for the content of the published article.

## Declaration of competing interest

The authors declare that they have no known competing financial interests or personal relationships that could have appeared to influence the work reported in this paper.

## Figures and Tables

**Appendix 1:** Differences in KAP scores among community pharmacists according to demographic and professional characteristics.

Variable	Knowledge median (min–max)	*p-value*	Attitude median (min–max)	*p-value*	Practice median (min–max)	*p-value*
Gender						
Female	17 (6–23)	1.000^a^	30 (8–32)	0.541^a^	118 (60–135)	0.688^a^
Male	16 (3−22)		30 (22−32)		119 (100−133)	
Age						
23–33	17 (10−23)	**< 0.001**^b^*	29 (8–32)	0.916^b^	118 (60–133)	0.598^b^
34–44	15 (3–22)		30 (13−32)		118 (101−130)	
45–55	16 (9–21)		30 (25–32)		120 (107–131)	
56–77	11 (7–20)		30 (24–32)		122.5 (99–135)	
Pharmacy location						
Malang Regency	16 (3–22)	0.057^a^	29 (8–32)	0.679^a^	118 (60–133)	0.649^a^
Malang City	17 (7–23)		30 (22–32)		118 (99–135)	
Educational level						
Licensed pharmacist	16 (3−23)	0.951^a^	30 (8–32)	0.370^a^	118 (60–135)	0.911^a^
Licensed pharmacist with a master's/doctoral degree	16.5 (9–22)		29 (24–32)		116.5 (99–131)	
Pharmacist position						
Pharmacist-in-charge	16 (3–23)	**0.004**^a^*	30 (8–32)	0.206^a^	118 (60–135)	0.809^a^
Supporting pharmacist	18 (11−23)		30 (26–32)		119 (104–130)	
Ownership status of the pharmacy						
Owned by the pharmacist practising in the pharmacy	16 (6–22)	**0.022**^a^*	29.5 (8–32)	0.835^a^	118.5 (60–131)	0.892^a^
Owned by another individual	17 (3–23)		30 (22–32)		118 (92–135)	
Pharmacy type						
Independent pharmacy	16 (6–22)	0.509^a^	29 (8–32)	0.212^a^	118 (60–135)	0.475^a^
Banner pharmacy	17 (3–23)		30 (23−32)		120.5 (99–130)	
Professional experience in community pharmacy practice						
1–6 months	18 (11−22)	**< 0.001**^b^*	30 (24–32)	0.471^b^	120 (101−133)	0.888^b^
7–11 months	17 (10−21)		29 (23–32)		115.5 (92–130)	
1–5 years	17 (7–23)		30 (22–32)		119 (99–132)	
6–10 years	16 (7–23)		30 (8–32)		117.5 (60–131)	
More than 10 years	15 (3–22)		30 (13–32)		115 (101–135)	
Average duration of daily practice						
≥ 5 h	17 (7–23)	0.292^a^	30 (8–32)	0.752^a^	119 (60–133)	0.280^a^
< 5 h	16 (3–22)		29 (13–32)		117 (92–135)	
Average weekly working hours in community pharmacy						
< 25 h	16 (3–22)	0.092^b^	29 (22–32)	0.543^b^	117 (92–135)	0.648^b^
25–35 h	16 (7–22)		30 (8–32)		118 (60–133)	
36–40 h	15 (6–22)		30 (13–32)		120 (100−131)	
>40 h	17 (8–23)		30 (24–32)		120 (101–130)	

a indicates the Mann–Whitney test, ^b^ indicates the Kruskal–Wallis test, and * *p*-value <0.05 was considered statistically significant.

**Appendix 2:** Practices of community pharmacists regarding the common cold and influenza syndrome (continued).

Statements	Strongly Disagree n (%)	Disagree n (%)	Agree n (%)	Strongly Agree n (%)	RII	SD	Rank	*p-value*
I was able to distinguish between the common cold and influenza based on the symptoms presented by the patient.	2 (0.9)	19 (8.6)	105 (47.5)	95 (43.0)	0.83	0.67	27	0.505
I asked patients about possible sources of viral transmission for the common cold and/or influenza.	4 (1.8)	26 (11.8)	92 (41.6)	99 (44.8)	0.82	0.74	30	0.768
I asked adult or elderly patients about their medical history, such as hypertension, stroke, diabetes mellitus, or hyperthyroidism.	1 (0.5)	8 (3.6)	75 (33.9)	137 (62.0)	0.89	0.59	14	0.928
I asked adult patients about any history of alcoholism.	9 (4.1)	40 (18.1)	84 (38.0)	88 (39.8)	0.78	0.85	33	0.337
I asked patients whether they had any history of drug allergies.	1 (0.5)	8 (3.6)	61 (27.6)	151 (68.3)	0.91	0.58	12	0.617
I asked patients whether they had any history of drug intolerance (adverse reactions to certain medications).	2 (0.9)	24 (10.9)	74 (33.5)	121 (54.8)	0.86	0.72	23	0.599
I checked whether the medications recommended for the patients' common cold and/or influenza were appropriate for their indications.	1 (0.5)	5 (2.3)	68 (30.8)	147 (66.5)	0.91	0.55	11	0.287
I asked patients whether they preferred medications for the common cold and/or influenza that caused sedation (drowsiness) or not.	2 (0.9)	10 (4.5)	72 (32.6)	137 (62.0)	0.89	0.63	15	0.630
I always provided oral decongestant preparations for patients complaining of nasal congestion, a runny nose, and sneezing.	3 (1.4)	21 (9.5)	95 (43.0)	102 (46.2)	0.83	0.71	28	0.710
I dispensed antibiotics to patients who complained of sore throat and hoarseness.	93 (42.1)	66 (29.9)	32 (14.5)	30 (13.6)	0.50	1.06	36	0.688
I provided paracetamol for patients with a history of gastritis who complained of fever or headache.	3 (1.4)	8 (3.6)	77 (34.8)	133 (60.2)	0.88	0.64	19	0.060
I provided dosing regimen limits for paracetamol to patients with a history of alcoholism who complained of fever or headache.	5 (2.3)	18 (8.1)	99 (44.8)	99 (44.8)	0.83	0.72	29	0.239
I always provided combination preparations as the leading choice for managing common cold and/or influenza.	1 (0.5)	27 (12.2)	109 (49.3)	84 (38.0)	0.81	0.68	31	0.077
I provided nasal drops to infants experiencing a runny nose or nasal congestion.	8 (3.6)	23 (10.4)	103 (46.6)	87 (39.4)	0.80	0.77	32	0.501
I advised patients to wash their hands with soap to prevent the transmission of the common cold and/or influenza.	1 (0.5)	15 (6.8)	72 (32.6)	133 (60.2)	0.88	0.64	19	0.867
I advised patients to wear surgical masks to prevent the transmission of the common cold and/or influenza.	2 (0.9)	15 (6.8)	75 (33.9)	129 (58.4)	0.87	0.66	22	0.746
I advised patients to wear N95 masks to prevent the transmission of the common cold and/or influenza.	29 (13.1)	74 (33.5)	72 (32.6)	46 (20.8)	0.65	0.96	35	0.048*
I advised patients to consult a general practitioner (GP) if a child continued to experience influenza for more than 10 days.	8 (3.6)	14 (6.3)	45 (20.4)	154 (69.7)	0.89	0.77	18	0.190
I advised patients to consult a general practitioner (GP) if an adult continued to experience influenza for more than 5 days.	1 (0.5)	14 (6.3)	62 (28.1)	144 (65.2)	0.89	0.63	15	0.136
I informed patients that paracetamol should be taken after meals.	13 (5.9)	43 (19.5)	69 (31.2)	96 (43.4)	0.78	0.92	34	0.348
I informed patients that medications containing oral decongestants should be taken after meals.	5 (2.3)	14 (6.3)	87 (39.4)	115 (52.0)	0.85	0.71	24	0.351
I always explained the possible side effects of medications for the common cold and/or influenza.	1 (0.5)	10 (4.5)	88 (39.8)	122 (55.2)	0.87	0.61	21	0.593
I informed patients that medications containing antihistamines always caused drowsiness as a side effect.	11 (5.0)	19 (8.6)	68 (30.8)	123 (55.7)	0.84	0.84	26	0.190
I informed patients that they should not exceed the recommended daily dosage of common cold and/or influenza medications even if symptoms persisted.	4 (1.8)	5 (2.3)	79 (35.7)	133 (60.2)	0.89	0.64	17	0.145
I advised patients to drink plenty of warm water during the common cold and/or influenza.	0 (0.0)	7 (3.2)	78 (35.3)	136 (61.5)	0.90	0.55	13	0.991
I advised patients to avoid beverages containing caffeine, such as coffee or tea, during the common cold and/or influenza.	4 (1.8)	22 (10.0)	89 (40.3)	106 (48.0)	0.84	0.73	25	0.442

The Kruskal–Wallis test was applied, with RII representing the relative importance index and SD representing the standard deviation, categorized according to the duration of professional experience. * p-value <0.05 was considered statistically significant.

**Appendix 3:** Classification of knowledge scores based on Bloom's cut-off criteria.

Knowledge category	Score range (%)	n = 221 (%)
Good knowledge	≥ 80	59 (26.7)
Moderate knowledge	60–79	112 (50.7)
Poor knowledge	< 60	50 (22.6)

Classification was performed using Bloom's cut-off criteria (good ≥80%, moderate 60–79%, poor <60%) applied to pharmacists' total knowledge scores on the 23-item questionnaire.^21^

## Data Availability

The anonymized dataset derived from the pharmacists' KAP questionnaire responses is available from the corresponding author on reasonable request.

## Appendix B Supplementary data

Supplementary material 1 Image 1

Supplementary material 2 Image 2
