# Supplementary material for: Community pharmacists' knowledge, attitudes, and practices toward self-medication for common cold and influenza: A COM-B model–based cross-sectional study
Source: Explor Res Clin Soc Pharm. 2026 Apr 2;23:100735. doi: 10.1016/j.rcsop.2026.100735 (PMC13103582; doi:10.1016/j.rcsop.2026.100735)
Supplement: Supplementary file 1 — Supplementary material 1 [file mmc1.docx]

**Supplementary File 1: Knowledge, attitude, and practice responses by duration of professional experience**

**Domain: Knowledge**

| **Item** | **1–6 months** | | **7–11 months** | | **1–5 years** | | **6–10 years** | | **> 10 years** | | ***p-value*** |
| --- | --- | --- | --- | --- | --- | --- | --- | --- | --- | --- | --- |
|  | **True**  **n (%)** | **False**  **n (%)** | **True**  **n (%)** | **False**  **n (%)** | **True**  **n (%)** | **False**  **n (%)** | **True**  **n (%)** | **False**  **n (%)** | **True**  **n (%)** | **False**  **n (%)** |  |
| K1 | 21 (91.3) | 2 (8.7) | 19 (95.0) | 1 (5.0) | 85 (89.5) | 10 (10.5) | 47 (90.4) | 5 (9.6) | 24 (77.4) | 7 (22.6) | 0.371^a^ |
| K2 | 22 (95.7) | 1 (4.3) | 17 (85.0) | 3 (15.0) | 92 (96.8) | 3 (3.2) | 48 (92.3) | 4 (7.7) | 30 (96.8) | 1 (3.2) | 0.228^a^ |
| K3 | 13 (56.5) | 10 (43.5) | 13 (65.0) | 7 (35.0) | 61 (64.2) | 34 (35.8) | 28 (53.8) | 24 (46.2) | 17 (54.8) | 14 (45.2) | 0.703^b^ |
| K4 | 5 (21.7) | 18 (78.3) | 5 (25.0) | 15 (75.0) | 22 (23.2) | 73 (76.8) | 9 (17.3) | 43 (82.7) | 5 (16.1) | 26 (83.9) | 0.854^b^ |
| K5 | 20 (87.0) | 3 (13.0) | 14 (70.0) | 6 (30.0) | 64 (67.4) | 31 (32.6) | 32 (61.5) | 20 (38.5) | 18 (58.1) | 13 (41.9) | 0.196^b^ |
| K6 | 7 (30.4) | 16 (69.6) | 7 (35.0) | 13 (65.0) | 23 (24.2) | 72 (75.8) | 12 (23.1) | 40 (76.9) | 6 (19.4) | 25 (80.6) | 0.718^b^ |
| K7 | 23 (100) | 0 (0) | 20 (100) | 0 (0) | 90 (94.7) | 5 (5.3) | 50 (96.2) | 2 (3.8) | 29 (93.5) | 2 (6.5) | 0.828^a^ |
| K8 | 20 (87.0) | 3 (13.0) | 17 (85.0) | 3 (15.0) | 66 (69.5) | 29 (30.5) | 36 (69.2) | 16 (30.8) | 22 (71.0) | 9 (29.0) | 0.313^b^ |
| K9 | 22 (95.7) | 1 (4.3) | 19 (95.0) | 1 (5.0) | 80 (84.2) | 15 (15.8) | 46 (88.5) | 6 (11.5) | 23 (74.2) | 8 (25.8) | 0.161^a^ |
| K10 | 21 (91.3) | 2 (8.7) | 17 (85.0) | 3 (15.0) | 77 (81.1) | 18 (18.9) | 33 (63.5) | 19 (36.5) | 19 (61.3) | 12 (38.7) | **0.011^b^*** |
| K11 | 13 (56.5) | 10 (43.5) | 8 (40.0) | 12 (60.0) | 36 (37.9) | 59 (62.1) | 19 (36.5) | 33 (63.5) | 8 (25.8) | 23 (74.2) | 0.250^b^ |
| K12 | 15 (65.2) | 8 (34.8) | 10 (50.0) | 10 (50.0) | 44 (46.3) | 51 (53.7) | 17 (32.7) | 35 (67.3) | 22 (71.0) | 9 (29.0) | **0.037^b^*** |
| K13 | 19 (82.6) | 4 (17.4) | 17 (85.0) | 3 (15.0) | 85 (89.5) | 10 (10.5) | 41 (78.8) | 11 (21.2) | 23 (74.2) | 8 (25.8) | 0.256^b^ |
| K14 | 22 (95.7) | 1 (4.3) | 18 (90.0) | 2 (10.0) | 88 (92.6) | 7 (7.4) | 41 (78.8) | 11 (21.2) | 23 (74.2) | 8 (25.8) | **0.019^a^*** |
| K15 | 18 (78.3) | 5 (21.7) | 14 (70.0) | 6 (30.0) | 72 (75.8) | 23 (24.2) | 36 (69.2) | 16 (30.8) | 19 (61.3) | 12 (38.7) | 0.538^b^ |
| K16 | 17 (73.9) | 6 (26.1) | 12 (60.0) | 8 (40.0) | 64 (67.4) | 31 (32.6) | 30 (57.7) | 22 (42.3) | 20 (64.5) | 11 (35.5) | 0.651^b^ |
| K17 | 17 (73.9) | 6 (26.1) | 17 (85.0) | 3 (15.0) | 83 (87.4) | 12 (12.6) | 39 (75.0) | 13 (25.0) | 17 (54.8) | 14 (45.2) | **0.003^b^*** |
| K18 | 17 (73.9) | 6 (26.1) | 13 (65.0) | 7 (35.0) | 70 (73.7) | 25 (26.3) | 34 (65.4) | 18 (34.6) | 20 (64.5) | 11 (35.5) | 0.741^b^ |
| K19 | 12 (52.2) | 11 (47.8) | 12 (60.0) | 8 (40.0) | 53 (55.8) | 42 (44.2) | 27 (51.9) | 25 (48.1) | 15 (48.4) | 16 (51.6) | 0.921^b^ |
| K20 | 20 (87.0) | 3 (13.0) | 14 (70.0) | 6 (30.0) | 64 (67.4) | 31 (32.6) | 36 (69.2) | 16 (30.8) | 12 (38.7) | 19 (61.3) | **0.004^b^*** |
| K21 | 22 (95.7) | 1 (4.3) | 20 (100) | 0 (0) | 90 (94.7) | 5 (5.3) | 46 (88.5) | 6 (11.5) | 21 (67.7) | 10 (32.3) | **< 0.001^a^*** |
| K22 | 21 (91.3) | 2 (8.7) | 18 (90.0) | 2 (10.0) | 87 (91.6) | 8 (8.4) | 43 (82.7) | 9 (17.3) | 24 (77.4) | 7 (22.6) | 0.226^a^ |
| **Item** | **1–6 months** | | **7–11 months** | | **1–5 years** | | **6–10 years** | | **> 10 years** | | ***p-value*** |
|  | **True**  **n (%)** | **False**  **n (%)** | **True**  **n (%)** | **False**  **n (%)** | **True**  **n (%)** | **False**  **n (%)** | **True**  **n (%)** | **False**  **n (%)** | **True**  **n (%)** | **False**  **n (%)** |  |
| K23 | 21 (91.3) | 2 (8.7) | 15 (75.0) | 5 (25.0) | 86 (90.5) | 9 (9.5) | 47 (90.4) | 5 (9.6) | 22 (71.0) | 9 (29.0) | **0.033^a^*** |

^a^ Fisher’s exact test was applied, categorized according to duration of professional experience. * p-value < 0.05 was considered statistically significant.

^b^ Pearson’s chi-square test was applied, categorized according to duration of professional experience. * p-value < 0.05 was considered statistically significant.

**Domain: Attitude**

| **Item** | **1–6 months** | | | | **7–11 months** | | | | **1–5 years** | | | | ***p-value*** |
| --- | --- | --- | --- | --- | --- | --- | --- | --- | --- | --- | --- | --- | --- |
|  | **Strongly disagree**  **n (%)** | **Disagree**  **n (%)** | **Agree**  **n (%)** | **Strongly agree**  **n (%)** | **Strongly disagree**  **n (%)** | **Disagree**  **n (%)** | **Agree**  **n (%)** | **Strongly agree**  **n (%)** | **Strongly disagree**  **n (%)** | **Disagree**  **n (%)** | **Agree**  **n (%)** | **Strongly agree**  **n (%)** |  |
| A1 | 0 (0) | 0 (0) | 8 (34.8) | 15 (65.2) | 0 (0) | 1 (5.0) | 12 (60.0) | 7 (35.0) | 0 (0) | 3 (3.2) | 26 (27.4) | 66 (69.5) | 0.065 |
| A2 | 0 (0) | 0 (0) | 3 (13.0) | 20 (87.0) | 0 (0) | 1 (5.0) | 3 (15.0) | 16 (80.0) | 0 (0) | 0 (0) | 13 (13.7) | 82 (86.3) | 0.488 |
| A3 | 0 (0) | 2 (8.7) | 6 (26.1) | 15 (65.2) | 0 (0) | 0 (0) | 10 (50.0) | 10 (50.0) | 0 (0) | 2 (2.1) | 50 (52.6) | 43 (45.3) | 0.163 |
| A4 | 0 (0) | 1 (4.3) | 6 (26.1) | 16 (69.6) | 0 (0) | 0 (0) | 6 (30.0) | 14 (70.0) | 0 (0) | 0 (0) | 16 (16.8) | 79 (83.2) | 0.541 |
| A5 | 0 (0) | 1 (4.3) | 5 (21.7) | 17 (73.9) | 0 (0) | 1 (5.0) | 5 (25.0) | 14 (70.0) | 0 (0) | 1 (1.1) | 20 (21.1) | 74 (77.9) | 0.528 |
| A6 | 0 (0) | 0 (0) | 5 (21.7) | 18 (78.3) | 0 (0) | 0 (0) | 7 (35.0) | 13 (65.0) | 0 (0) | 0 (0) | 17 (17.9) | 78 (82.1) | 0.269 |
| A7 | 0 (0) | 0 (0) | 6 (26.1) | 17 (73.9) | 0 (0) | 1 (5.0) | 8 (40.0) | 11 (55.0) | 0 (0) | 1 (1.1) | 26 (27.4) | 68 (71.6) | 0.548 |
| A8 | 0 (0) | 0 (0) | 4 (17.4) | 19 (82.6) | 0 (0) | 0 (0) | 4 (20.0) | 16 (80.0) | 0 (0) | 0 (0) | 20 (21.1) | 75 (78.9) | 0.651 |

| **Item** | **6–10 years** | | | | **> 10 years** | | | | ***p-value*** |
| --- | --- | --- | --- | --- | --- | --- | --- | --- | --- |
|  | **Strongly disagree**  **n (%)** | **Disagree**  **n (%)** | **Agree**  **n (%)** | **Strongly agree**  **n (%)** | **Strongly disagree**  **n (%)** | **Disagree**  **n (%)** | **Agree**  **n (%)** | **Strongly agree**  **n (%)** |  |
| A1 | 1 (1.9) | 1 (1.9) | 14 (26.9) | 36 (69.2) | 1 (3.2) | 1 (3.2) | 10 (32.3) | 19 (61.3) | 0.065 |
| A2 | 1 (1.9) | 0 (0) | 6 (11.5) | 45 (86.5) | 1 (3.2) | 0 (0) | 7 (22.6) | 23 (74.2) | 0.488 |
| A3 | 1 (1.9) | 1 (1.9) | 17 (32.7) | 33 (63.5) | 1 (3.2) | 1 (3.2) | 9 (29.0) | 20 (64.5) | 0.163 |
| A4 | 1 (1.9) | 1 (1.9) | 14 (26.9) | 36 (69.2) | 1 (3.2) | 0 (0) | 8 (25.8) | 22 (71.0) | 0.541 |
| A5 | 1 (1.9) | 1 (1.9) | 13 (25.0) | 37 (71.2) | 1 (3.2) | 1 (3.2) | 7 (22.6) | 22 (71.0) | 0.528 |
| **Item** | **6–10 years** | | | | **> 10 years** | | | | ***p-value*** |
|  | **Strongly disagree**  **n (%)** | **Disagree**  **n (%)** | **Agree**  **n (%)** | **Strongly agree**  **n (%)** | **Strongly disagree**  **n (%)** | **Disagree**  **n (%)** | **Agree**  **n (%)** | **Strongly agree**  **n (%)** |  |
| A6 | 1 (1.9) | 1 (1.9) | 14 (26.9) | 36 (69.2) | 1 (3.2) | 0 (0) | 7 (22.6) | 23 (74.2) | 0.269 |
| A7 | 1 (1.9) | 2 (3.8) | 15 (28.8) | 34 (65.4) | 0 (0) | 1 (3.2) | 9 (29.0) | 21 (67.7) | 0.548 |
| A8 | 1 (1.9) | 0 (0) | 10 (19.2) | 41 (78.8) | 0 (0) | 1 (3.2) | 9 (29.0) | 21 (67.7) | 0.651 |

The Kruskal–Wallis test was applied, categorized according to the duration of professional experience. * p-value < 0.05 was considered statistically significant.

**Domain: Practice**

| **Item** | **1–6 months** | | | | **7–11 months** | | | | **1–5 years** | | | | ***p-value*** |
| --- | --- | --- | --- | --- | --- | --- | --- | --- | --- | --- | --- | --- | --- |
|  | **Strongly disagree**  **n (%)** | **Disagree**  **n (%)** | **Agree**  **n (%)** | **Strongly agree**  **n (%)** | **Strongly disagree**  **n (%)** | **Disagree**  **n (%)** | **Agree**  **n (%)** | **Strongly agree**  **n (%)** | **Strongly disagree**  **n (%)** | **Disagree**  **n (%)** | **Agree**  **n (%)** | **Strongly agree**  **n (%)** |  |
| P1 | 0 (0) | 0 (0) | 4 (17.4) | 19 (82.6) | 0 (0) | 0 (0) | 4 (20.0) | 16 (80.0) | 0 (0) | 0 (0) | 15 (15.8) | 80 (84.2) | 0.435 |
| P2 | 0 (0) | 0 (0) | 4 (17.4) | 19 (82.6) | 0 (0) | 0 (0) | 4 (20.0) | 16 (80.0) | 0 (0) | 0 (0) | 15 (15.8) | 80 (84.2) | 0.618 |
| P3 | 0 (0) | 0 (0) | 4 (17.4) | 19 (82.6) | 0 (0) | 0 (0) | 3 (15.0) | 17 (85.0) | 0 (0) | 4 (4.2) | 23 (24.2) | 68 (71.6) | 0.350 |
| P4 | 0 (0) | 1 (4.3) | 5 (21.7) | 17 (73.9) | 0 (0) | 1 (5.0) | 4 (20.0) | 15 (75.0) | 0 (0) | 4 (4.2) | 18 (18.9) | 73 (76.8) | 0.505 |
| P5 | 0 (0) | 2 (8.7) | 6 (26.1) | 15 (65.2) | 0 (0) | 2 (10.0) | 5 (25.0) | 13 (65.0) | 0 (0) | 4 (4.2) | 26 (27.4) | 65 (68.4) | 0.768 |
| P6 | 0 (0) | 1 (4.3) | 6 (26.1) | 16 (69.6) | 0 (0) | 1 (5.0) | 3 (15.0) | 16 (80.0) | 0 (0) | 1 (1.1) | 22 (23.2) | 72 (75.8) | 0.432 |
| P7 | 0 (0) | 0 (0) | 6 (26.1) | 17 (73.9) | 0 (0) | 1 (5.0) | 8 (40.0) | 11 (55.0) | 0 (0) | 0 (0) | 22 (23.2) | 73 (76.8) | 0.928 |
| P8 | 0 (0) | 0 (0) | 4 (17.4) | 19 (82.6) | 0 (0) | 0 (0) | 4 (20.0) | 16 (80.0) | 0 (0) | 0 (0) | 17 (17.9) | 78 (82.1) | 0.870 |
| P9 | 0 (0) | 0 (0) | 4 (17.4) | 19 (82.6) | 0 (0) | 0 (0) | 4 (20.0) | 16 (80.0) | 0 (0) | 1 (1.1) | 23 (24.2) | 71 (74.7) | 0.087 |
| P10 | 0 (0) | 1 (4.3) | 4 (17.4) | 18 (78.3) | 0 (0) | 1 (5.0) | 6 (30.0) | 13 (65.0) | 0 (0) | 3 (3.2) | 22 (23.2) | 70 (73.7) | 0.337 |
| P11 | 0 (0) | 1 (4.3) | 5 (21.7) | 17 (73.9) | 0 (0) | 1 (5.0) | 4 (20.0) | 15 (75.0) | 0 (0) | 1 (1.1) | 22 (23.2) | 72 (75.8) | 0.617 |
| P12 | 0 (0) | 1 (4.3) | 5 (21.7) | 17 (73.9) | 0 (0) | 0 (0) | 6 (30.0) | 14 (70.0) | 0 (0) | 2 (2.1) | 18 (18.9) | 75 (78.9) | 0.599 |
| P13 | 0 (0) | 2 (8.7) | 7 (30.4) | 14 (60.9) | 0 (0) | 0 (0) | 4 (20.0) | 16 (80.0) | 0 (0) | 4 (4.2) | 35 (36.8) | 56 (58.9) | 0.287 |
| P14 | 0 (0) | 1 (4.3) | 6 (26.1) | 16 (69.6) | 0 (0) | 1 (5.0) | 4 (20.0) | 15 (75.0) | 0 (0) | 3 (3.2) | 19 (20.0) | 73 (76.8) | 0.912 |
| P15 | 0 (0) | 1 (4.3) | 5 (21.7) | 17 (73.9) | 0 (0) | 1 (5.0) | 5 (25.0) | 14 (70.0) | 0 (0) | 2 (2.1) | 20 (21.1) | 73 (76.8) | 0.630 |
| P16 | 0 (0) | 1 (4.3) | 5 (21.7) | 17 (73.9) | 0 (0) | 0 (0) | 5 (25.0) | 15 (75.0) | 0 (0) | 3 (3.2) | 20 (21.1) | 72 (75.8) | 0.710 |
| P17 | 0 (0) | 0 (0) | 6 (26.1) | 17 (73.9) | 0 (0) | 0 (0) | 7 (35.0) | 13 (65.0) | 0 (0) | 0 (0) | 21 (22.1) | 74 (77.9) | 0.688 |
| **Item** | **1–6 months** | | | | **7–11 months** | | | | **1–5 years** | | | | ***p-value*** |
|  | **Strongly disagree**  **n (%)** | **Disagree**  **n (%)** | **Agree**  **n (%)** | **Strongly agree**  **n (%)** | **Strongly disagree**  **n (%)** | **Disagree**  **n (%)** | **Agree**  **n (%)** | **Strongly agree**  **n (%)** | **Strongly disagree**  **n (%)** | **Disagree**  **n (%)** | **Agree**  **n (%)** | **Strongly agree**  **n (%)** |  |
| P18 | 0 (0) | 0 (0) | 6 (26.1) | 17 (73.9) | 0 (0) | 0 (0) | 6 (30.0) | 14 (70.0) | 0 (0) | 1 (1.1) | 19 (20.0) | 75 (78.9) | 0.060 |
| P19 | 0 (0) | 0 (0) | 5 (21.7) | 18 (78.3) | 0 (0) | 1 (5.0) | 5 (25.0) | 14 (70.0) | 0 (0) | 1 (1.1) | 21 (22.1) | 73 (76.8) | 0.239 |
| P20 | 0 (0) | 0 (0) | 4 (17.4) | 19 (82.6) | 0 (0) | 0 (0) | 4 (20.0) | 16 (80.0) | 0 (0) | 1 (1.1) | 23 (24.2) | 71 (74.7) | 0.077 |
| P21 | 0 (0) | 1 (4.3) | 5 (21.7) | 17 (73.9) | 0 (0) | 0 (0) | 6 (30.0) | 14 (70.0) | 0 (0) | 1 (1.1) | 21 (22.1) | 73 (76.8) | 0.501 |
| P22 | 0 (0) | 1 (4.3) | 6 (26.1) | 16 (69.6) | 0 (0) | 1 (5.0) | 4 (20.0) | 15 (75.0) | 0 (0) | 1 (1.1) | 22 (23.2) | 72 (75.8) | 0.867 |
| P23 | 0 (0) | 2 (8.7) | 7 (30.4) | 14 (60.9) | 0 (0) | 1 (5.0) | 5 (25.0) | 14 (70.0) | 0 (0) | 5 (5.3) | 40 (42.1) | 50 (52.6) | 0.746 |
| P24 | 0 (0) | 1 (4.3) | 5 (21.7) | 17 (73.9) | 0 (0) | 1 (5.0) | 5 (25.0) | 14 (70.0) | 0 (0) | 1 (1.1) | 19 (20.0) | 75 (78.9) | **0.048*** |
| P25 | 0 (0) | 1 (4.3) | 6 (26.1) | 16 (69.6) | 0 (0) | 1 (5.0) | 4 (20.0) | 15 (75.0) | 0 (0) | 2 (2.1) | 18 (18.9) | 75 (78.9) | 0.190 |
| P26 | 0 (0) | 2 (8.7) | 7 (30.4) | 14 (60.9) | 0 (0) | 1 (5.0) | 6 (30.0) | 13 (65.0) | 0 (0) | 5 (5.3) | 33 (34.7) | 57 (60.0) | 0.136 |
| P27 | 0 (0) | 1 (4.3) | 9 (39.1) | 13 (56.5) | 0 (0) | 1 (5.0) | 6 (30.0) | 13 (65.0) | 0 (0) | 6 (6.3) | 38 (40.0) | 51 (53.7) | 0.348 |
| P28 | 0 (0) | 1 (4.3) | 7 (30.4) | 15 (65.2) | 0 (0) | 2 (10.0) | 6 (30.0) | 12 (60.0) | 0 (0) | 7 (7.4) | 36 (37.9) | 52 (54.7) | 0.363 |
| P29 | 0 (0) | 1 (4.3) | 4 (17.4) | 18 (78.3) | 0 (0) | 2 (10.0) | 4 (20.0) | 14 (70.0) | 0 (0) | 6 (6.3) | 22 (23.2) | 67 (70.5) | 0.351 |
| P30 | 0 (0) | 1 (4.3) | 6 (26.1) | 16 (69.6) | 0 (0) | 2 (10.0) | 5 (25.0) | 13 (65.0) | 0 (0) | 3 (3.2) | 23 (24.2) | 69 (72.6) | 0.593 |
| P31 | 1 (4.3) | 1 (4.3) | 7 (30.4) | 14 (60.9) | 0 (0) | 2 (10.0) | 4 (20.0) | 14 (70.0) | 1 (1.1) | 3 (3.2) | 22 (23.2) | 69 (72.6) | 0.190 |
| P32 | 0 (0) | 0 (0) | 4 (17.4) | 19 (82.6) | 0 (0) | 0 (0) | 4 (20.0) | 16 (80.0) | 0 (0) | 0 (0) | 20 (21.1) | 75 (78.9) | 0.145 |
| P33 | 0 (0) | 1 (4.3) | 8 (34.8) | 14 (60.9) | 0 (0) | 1 (5.0) | 6 (30.0) | 13 (65.0) | 0 (0) | 2 (2.1) | 26 (27.4) | 67 (70.5) | 0.991 |
| P34 | 0 (0) | 0 (0) | 4 (17.4) | 19 (82.6) | 0 (0) | 0 (0) | 4 (20.0) | 16 (80.0) | 0 (0) | 0 (0) | 20 (21.1) | 75 (78.9) | 0.442 |
| P35 | 0 (0) | 1 (4.3) | 7 (30.4) | 15 (65.2) | 0 (0) | 1 (5.0) | 6 (30.0) | 13 (65.0) | 0 (0) | 1 (1.1) | 22 (23.2) | 72 (75.8) | 0.216 |
| P36 | 0 (0) | 0 (0) | 6 (26.1) | 17 (73.9) | 0 (0) | 1 (5.0) | 6 (30.0) | 13 (65.0) | 0 (0) | 2 (2.1) | 20 (21.1) | 73 (76.8) | 0.119 |

| **Item** | **6–10 years** | | | | **> 10 years** | | | | ***p-value*** |
| --- | --- | --- | --- | --- | --- | --- | --- | --- | --- |
|  | **Strongly disagree**  **n (%)** | **Disagree**  **n (%)** | **Agree**  **n (%)** | **Strongly agree**  **n (%)** | **Strongly disagree**  **n (%)** | **Disagree**  **n (%)** | **Agree**  **n (%)** | **Strongly agree**  **n (%)** |  |
| P1 | 0 (0) | 0 (0) | 11 (21.2) | 41 (78.8) | 0 (0) | 0 (0) | 6 (19.4) | 15 (80.6) | 0.435 |
| P2 | 0 (0) | 0 (0) | 11 (21.2) | 41 (78.8) | 0 (0) | 0 (0) | 6 (19.4) | 25 (80.6) | 0.618 |
| P3 | 0 (0) | 2 (3.8) | 15 (28.8) | 35 (67.3) | 1 (3.2) | 1 (3.2) | 10 (32.3) | 19 (61.3) | 0.350 |
| P4 | 1 (1.9) | 2 (3.8) | 12 (23.1) | 37 (71.2) | 1 (3.2) | 1 (3.2) | 8 (25.8) | 21 (67.7) | 0.505 |
| P5 | 1 (1.9) | 3 (5.8) | 15 (28.8) | 33 (63.5) | 1 (3.2) | 2 (6.5) | 9 (29.0) | 19 (61.3) | 0.768 |
| P6 | 1 (1.9) | 2 (3.8) | 13 (25.0) | 36 (69.2) | 1 (3.2) | 1 (3.2) | 8 (25.8) | 21 (67.7) | 0.432 |
| P7 | 1 (1.9) | 1 (1.9) | 20 (38.5) | 30 (57.7) | 1 (3.2) | 0 (0) | 7 (22.6) | 23 (74.2) | 0.928 |
| P8 | 1 (1.9) | 0 (0) | 10 (19.2) | 41 (78.8) | 0 (0) | 1 (3.2) | 7 (22.6) | 23 (74.2) | 0.870 |
| P9 | 1 (1.9) | 1 (1.9) | 14 (26.9) | 26 (69.2) | 0 (0) | 1 (3.2) | 8 (25.8) | 22 (71.0) | 0.087 |
| P10 | 0 (0) | 3 (5.8) | 16 (30.8) | 33 (63.5) | 1 (3.2) | 1 (3.2) | 7 (22.6) | 22 (71.0) | 0.337 |
| P11 | 1 (1.9) | 2 (3.8) | 12 (23.1) | 37 (71.2) | 1 (3.2) | 1 (3.2) | 8 (25.8) | 21 (67.7) | 0.617 |
| P12 | 1 (1.9) | 1 (1.9) | 13 (25.0) | 37 (71.2) | 1 (3.2) | 1 (3.2) | 7 (22.6) | 22 (71.0) | 0.599 |
| P13 | 1 (1.9) | 3 (5.8) | 20 (38.5) | 28 (53.8) | 1 (3.2) | 1 (3.2) | 11 (35.5) | 18 (58.1) | 0.287 |
| P14 | 1 (1.9) | 2 (3.8) | 13 (25.0) | 36 (69.2) | 1 (3.2) | 1 (3.2) | 8 (25.8) | 21 (67.7) | 0.912 |
| P15 | 1 (1.9) | 1 (1.9) | 13 (25.0) | 37 (71.2) | 1 (3.2) | 1 (3.2) | 8 (25.8) | 21 (67.7) | 0.630 |
| P16 | 1 (1.9) | 1 (1.9) | 12 (23.1) | 38 (73.1) | 1 (3.2) | 1 (3.2) | 8 (25.8) | 21 (67.7) | 0.710 |
| P17 | 1 (1.9) | 2 (3.8) | 15 (28.8) | 34 (65.4) | 1 (3.2) | 0 (0) | 7 (22.6) | 23 (74.2) | 0.688 |
| P18 | 1 (1.9) | 1 (1.9) | 13 (25.0) | 37 (71.2) | 1 (3.2) | 0 (0) | 8 (25.8) | 22 (71.0) | 0.060 |
| P19 | 1 (1.9) | 1 (1.9) | 13 (25.0) | 37 (71.2) | 1 (3.2) | 1 (3.2) | 7 (22.6) | 22 (71.0) | 0.239 |
| P20 | 1 (1.9) | 1 (1.9) | 14 (26.9) | 36 (69.2) | 0 (0) | 1 (3.2) | 8 (25.8) | 22 (71.0) | 0.077 |
| P21 | 1 (1.9) | 1 (1.9) | 13 (25.0) | 37 (71.2) | 1 (3.2) | 1 (3.2) | 7 (22.6) | 22 (71.0) | 0.501 |
| P22 | 1 (1.9) | 2 (3.8) | 12 (23.1) | 37 (71.2) | 1 (3.2) | 1 (3.2) | 8 (25.8) | 21 (67.7) | 0.867 |
| P23 | 1 (1.9) | 4 (7.7) | 21 (40.4) | 26 (50.0) | 2 (6.5) | 3 (9.7) | 12 (38.7) | 14 (45.2) | 0.746 |
| P24 | 1 (1.9) | 2 (3.8) | 13 (25.0) | 36 (69.2) | 1 (3.2) | 1 (3.2) | 7 (22.6) | 22 (71.0) | **0.048*** |
| P25 | 1 (1.9) | 1 (1.9) | 13 (25.0) | 37 (71.2) | 1 (3.2) | 1 (3.2) | 7 (22.6) | 22 (71.0) | 0.190 |
| P26 | 1 (1.9) | 2 (3.8) | 18 (34.6) | 31 (59.6) | 1 (3.2) | 3 (9.7) | 12 (38.7) | 15 (48.4) | 0.136 |
| **Item** | **6–10 years** | | | | **> 10 years** | | | | ***p-value*** |
|  | **Strongly disagree**  **n (%)** | **Disagree**  **n (%)** | **Agree**  **n (%)** | **Strongly agree**  **n (%)** | **Strongly disagree**  **n (%)** | **Disagree**  **n (%)** | **Agree**  **n (%)** | **Strongly agree**  **n (%)** |  |
| P27 | 1 (1.9) | 5 (9.6) | 22 (42.3) | 24 (46.2) | 2 (6.5) | 4 (12.9) | 12 (38.7) | 13 (41.9) | 0.348 |
| P28 | 1 (1.9) | 7 (13.5) | 21 (40.4) | 23 (44.2) | 3 (9.7) | 4 (12.9) | 13 (41.9) | 11 (35.5) | 0.363 |
| P29 | 1 (1.9) | 7 (13.5) | 18 (34.6) | 26 (50.0) | 2 (6.5) | 3 (9.7) | 8 (25.8) | 18 (58.1) | 0.351 |
| P30 | 1 (1.9) | 3 (5.8) | 16 (30.8) | 32 (61.5) | 1 (3.2) | 3 (9.7) | 10 (32.3) | 17 (54.8) | 0.593 |
| P31 | 1 (1.9) | 3 (5.8) | 16 (30.8) | 32 (61.5) | 1 (3.2) | 3 (9.7) | 10 (32.3) | 17 (54.8) | 0.190 |
| P32 | 0 (0) | 0 (0) | 10 (19.2) | 42 (80.8) | 0 (0) | 0 (0) | 9 (29.0) | 22 (71.0) | 0.145 |
| P33 | 1 (1.9) | 3 (5.8) | 17 (32.7) | 31 (59.6) | 1 (3.2) | 2 (6.5) | 11 (35.5) | 17 (54.8) | 0.991 |
| P34 | 1 (1.9) | 0 (0) | 10 (19.2) | 41 (78.8) | 1 (3.2) | 1 (3.2) | 9 (29.0) | 20 (64.5) | 0.442 |
| P35 | 1 (1.9) | 2 (3.8) | 15 (28.8) | 34 (65.4) | 1 (3.2) | 1 (3.2) | 9 (29.0) | 20 (64.5) | 0.216 |
| P36 | 1 (1.9) | 1 (1.9) | 14 (26.9) | 36 (69.2) | 1 (3.2) | 1 (3.2) | 8 (25.8) | 21 (67.7) | 0.119 |

The Kruskal–Wallis test was applied, categorized according to the duration of professional experience. * p-value < 0.05 was considered statistically significant.

**Domain: Knowledge**

| **Item** | **Questions** |
| --- | --- |
| K1 | What is the pathogen that causes the common cold syndrome? |
| K2 | What is the pathogen that causes the influenza syndrome? |
| K3 | What are the symptoms of the common cold syndrome? |
| K4 | What are the symptoms of the influenza syndrome? |
| K5 | Which cold medication is safe for pregnant women? |
| K6 | Which cold medication is safe for breastfeeding women? |
| K7 | What medication is used to relieve muscle pain caused by influenza? |
| K8 | What medication is used to relieve muscle pain due to influenza in patients with a history of asthma? |
| K9 | What medication is used to treat cough in patients who require a mucolytic? |
| K10 | What medication is used to relieve nasal congestion in patients with uncontrolled hypertension? |
| K11 | Which of the following comorbid conditions, namely chronic bronchitis, hyperthyroidism, lung cancer, or chronic kidney disease, should be considered by pharmacists when selecting oral decongestants for patients with common cold syndrome? |
| K12 | What is the benefit of administering zinc supplements to patients with the common cold and/or influenza syndrome? |
| K13 | What is the class of drugs that work by breaking the protein bonds in mucus to facilitate sputum expulsion? |
| K14 | What is the class of drugs that act by stimulating alpha-1 adrenergic receptors? |
| K15 | What is the mechanism of action of decongestant drugs? |
| K16 | What is the recommended daily dose (mg) of vitamin C after the onset of symptoms to reduce the severity and duration of common cold and/or influenza symptoms? |
| K17 | What is the maximum daily dose (g) of paracetamol for fever in adults without a history of alcoholism? |
| K18 | What is the maximum daily dose (g) of paracetamol for fever in adults with a history of alcoholism? |
| K19 | What is the maximum daily dose (g) of paracetamol for fever in children aged 8–10 years? |
| K20 | Which medication is contraindicated for an 8-year-old child with a fever due to influenza? |
| K21 | Which medication may cause Reye’s syndrome if given to a child with influenza? |
| K22 | Reye’s syndrome is a serious condition that can cause organ damage. Which organ is affected? |
| K23 | What short-term side effect may occur after administering ibuprofen to relieve fever in adults or children? |

**Domain: Attitude**

| **Item** | **Statements** |
| --- | --- |
| A1 | I used the latest and most relevant references as guidance when providing information about medications for the common cold and/or influenza. |
| A2 | I ensured the accuracy of the information provided to patients regarding the use of medications for the common cold and/or influenza. |
| A3 | I needed self-medication consultation support tools (such as brochures or health applications) to provide information about common cold and/or influenza medications to patients. |
| A4 | I actively participated in seminars, webinars, and interprofessional discussions to increase my knowledge about managing common cold and/or influenza syndromes. |
| A5 | I demonstrated confidence when providing self-medication consultation services to patients regarding to the management of common colds and/or influenza. |
| A6 | I provided self-medication services for the common cold and/or influenza in a rational manner. |
| A7 | I always explained the directions for using medications for the common cold and/or influenza to patients in a clear and concise manner. |
| A8 | I always provided patients with information on how to use medications for common colds and/or influenza politely and respectfully. |

**Domain: Practice**

| **Item** | **Statements** |
| --- | --- |
| P1 | I conducted patient assessments (gathering information related to the patient’s condition) to determine the appropriate medication for the common cold and/or influenza. |
| P2 | I asked patients about the symptoms they experienced. |
| P3 | I asked patients about the duration of their symptoms. |
| P4 | I was able to distinguish between the common cold and influenza based on the symptoms presented by the patient. |
| P5 | I asked patients about possible sources of viral transmission for the common cold and/or influenza. |
| P6 | I asked patients about their age. |
| P7 | I asked adult or elderly patients about their medical history, such as hypertension, stroke, diabetes mellitus, or hyperthyroidism. |
| P8 | I asked female adult patients whether they were pregnant or breastfeeding. |
| P9 | I asked patients whether they were currently using or had recently used medications for the common cold and/or influenza. |
| P10 | I asked adult patients about any history of alcoholism. |
| P11 | I asked patients whether they had any history of drug allergies. |
| P12 | I asked patients whether they had any history of drug intolerance (adverse reactions to certain medications). |
| P13 | I checked whether the medications recommended for the patients’ common cold and/or influenza were appropriate for their indications. |
| P14 | I checked the accuracy of the dosage and administration of medications for the common cold and/or influenza before dispensing them to children. |
| P15 | I asked patients whether they preferred medications for the common cold and/or influenza that caused sedation (drowsiness) or not. |
| P16 | I always provided oral decongestant preparations for patients complaining of nasal congestion, a runny nose, and sneezing. |
| P17 | I dispensed antibiotics to patients who complained of sore throat and hoarseness. |
| P18 | I provided paracetamol for patients with a history of gastritis who complained of fever or headache. |
| P19 | I provided dosing regimen limits for paracetamol to patients with a history of alcoholism who complained of fever or headache. |
| P20 | I always provided combination preparations as the leading choice for managing common cold and/or influenza. |
| P21 | I provided nasal drops to infants experiencing a runny nose or nasal congestion. |
| P22 | I advised patients to wash their hands with soap to prevent the transmission of the common cold and/or influenza. |
| P23 | I advised patients to wear surgical masks to prevent the transmission of the common cold and/or influenza. |
| P24 | I advised patients to wear N95 masks to prevent the transmission of the common cold and/or influenza. |
| P25 | I advised patients to consult a general practitioner (GP) if a child continued to experience influenza for more than 10 days. |
| P26 | I advised patients to consult a general practitioner (GP) if an adult continued to experience influenza for more than 5 days. |
| P27 | I informed patients that paracetamol should be taken after meals. |
| P28 | I informed patients that ibuprofen should be taken after meals. |
| P29 | I informed patients that medications containing oral decongestants should be taken after meals. |
| P30 | I always explained the possible side effects of medications for the common cold and/or influenza. |
| P31 | I informed patients that medications containing antihistamines always caused drowsiness as a side effect. |
| P32 | I informed patients that they should not exceed the recommended daily dosage of common cold and/or influenza medications even if symptoms persisted. |
| P33 | I advised patients to drink plenty of warm water during the common cold and/or influenza. |
| P34 | I advised patients to avoid beverages containing caffeine, such as coffee or tea, during the common cold and/or influenza. |
| P35 | I advised patients to get adequate rest and sleep during the common cold and/or influenza. |
| P36 | I advised patients to avoid respiratory irritants, such as smoke, air pollution, and dust, during the common cold and/or influenza. |
