# Supplementary material for: Community pharmacists' knowledge, attitudes, and practices toward self-medication for common cold and influenza: A COM-B model–based cross-sectional study
Source: Explor Res Clin Soc Pharm. 2026 Apr 2;23:100735. doi: 10.1016/j.rcsop.2026.100735 (PMC13103582; doi:10.1016/j.rcsop.2026.100735)
Supplement: Supplementary file 2 — Supplementary material 2 [file mmc2.docx]

**Supplementary File 2: STROBE checklist**

The STROBE Statement—Checklist of Items That Should Be Addressed in Reports of Observational Studies

Developed from:

Vandenbroucke JP, von Elm E, Altman DG, Gøtzsche PC, Mulrow CD, Pocock SJ, Poole C, Schlesselman JJ, Egger M. Strengthening the reporting of observational studies in epidemiology (STROBE): explanation and elaboration. PLoS Medicine. 2007. Volume 4, Number 10: pp. 1628–1654. <https://doi.org/10.1371/journal.pmed.0040297>.

| **Item Number** | **Reporting Item** | **Reported on Page #** |
| --- | --- | --- |
| **TITLE AND ABSTRACT** | | |
| 1a. Title | Indicate the study’s design with a commonly used term in the title or the abstract. | Anonymized manuscript:  Pg 1 |
|  | **“Community pharmacists’ … : A COM-B model–based cross-sectional study”** |  |
| 1b. Abstract | Provide in the abstract an informative and balanced summary of what was done and what was found. | Anonymized manuscript:  Pg 1 |
|  | “**Methods: A cross-sectional survey was conducted among 221 community pharmacists …**” and  “**Results: … Attitude was the strongest predictor of practice …**” |  |
| **INTRODUCTION** | | |
| 2. Background/rationale | Explain the scientific background and rationale for the investigation being reported. | Anonymized manuscript:  Pg 2–3 |
|  | **“Common cold and influenza are widely self-managed using OTC medicines in many LMICs, including Indonesia; however, inadequate guidance may lead to inappropriate medicine use. Community pharmacists play a key role in responsible self-medication, although variations in consultation quality and the behavioral determinants underlying these practices remain insufficiently explored.”** |  |
| 3. Objectives | State specific objectives, including any prespecified hypotheses. | Anonymized manuscript:  Pg 3 |
|  | **“… this study aimed to evaluate community pharmacists’ knowledge, attitudes, and practices regarding self-medication for the common cold and influenza, and to analyze behavioral determinants influencing their practice using the COM-B model.”** |  |
| **METHODS** | | |
| 4. Study design | Present key elements of study design early in the paper. | Anonymized manuscript:  Pg 3 |
|  | **“A quantitative cross-sectional survey was conducted … to assess community pharmacists’ knowledge, attitudes, and practices (KAP) related to self-medication consultations for the common cold and influenza.”** |  |
| 5. Setting | Describe the setting, locations, and relevant dates, including periods of recruitment, exposure, follow-up, and data collection. | Anonymized manuscript:  Pg 3 |
|  | **“… community pharmacists working in retail pharmacies across the Malang Region, East Java, Indonesia … including Malang City and Malang Regency. Data collection was conducted between September and October 2025.”** |  |
| 6a. Participants | Cross-sectional study—Give the eligibility criteria, and the sources and methods of selection of participants. | Anonymized manuscript:  Pg 3 |
|  | **“… community pharmacists practicing in the Malang Region were recruited using convenience and snowball sampling … eligible participants were licensed pharmacists providing pharmaceutical services in community pharmacies who completed the questionnaire and provided informed consent.”** |  |
| 7. Variables | Clearly define all outcomes, exposures, predictors, potential confounders, and effect modifiers. Give diagnostic criteria, if applicable. | Anonymized manuscript:  Pg 3–5 |
|  | **“… knowledge, attitudes, and practices (KAP) regarding self-medication consultations were assessed using a structured questionnaire … with knowledge representing psychological capability, attitudes reflecting motivation, and practice corresponding to behavioral performance within the COM-B framework.”** |  |
| 8. Data sources/measurement | For each variable of interest, give sources of data and details of methods of assessment (measurement). Describe comparability of assessment methods if there is more than one group. | Anonymized manuscript:  Pg 4–5 |
|  | **“… a 67-item KAP questionnaire developed from literature and consultation guidelines … content validity assessed by experts, face validity and reliability tested among pharmacists … with knowledge scored as correct/incorrect and attitude and practice measured using four-point Likert scales.”** |  |
| 9. Bias | Describe any efforts to address potential sources of bias. | Anonymized manuscript:  Pg 4–5 |
|  | **“… instrument validity and reliability were established through expert review and pilot testing … and responses were screened for completeness before statistical analysis.”** |  |
| 10. Study size | Explain how the study size was arrived at. | Anonymized manuscript:  Pg 3 |
|  | **“… the minimum sample size was calculated as 217 pharmacists from a population of 473 using the Slovin formula with a 5% margin of error.”** |  |
| 11. Quantitative variables | Explain how quantitative variables were handled in the analyses. If applicable, describe which groupings were chosen, and why. | Anonymized manuscript:  Pg 4 |
|  | **“… knowledge items were scored as correct or incorrect, while attitude and practice items were measured using four-point Likert scales … knowledge scores were categorized using Bloom’s cut-off criteria (good, moderate, poor).”** |  |
| 12a. Statistical methods | Describe all statistical methods, including those used to control for confounding. | Anonymized manuscript:  Pg 4–5 |
|  | **“… descriptive statistics summarized participant characteristics and KAP scores … Pearson’s chi-square or Fisher’s exact tests, Kruskal–Wallis tests, item difficulty index (Pd), and relative importance index (RII) were applied … multiple linear regression was used to identify predictors of KAP scores.”** |  |
| 12b. Statistical methods | Describe any methods used to examine subgroups and interactions. | Anonymized manuscript:  Pg 5 |
|  | **“… Mann–Whitney U and Kruskal–Wallis tests were used to compare KAP scores across demographic and professional subgroups.”** |  |
| 12c. Statistical methods | Explain how missing data were addressed | Anonymized manuscript:  Pg 3–4 |
|  | **“… incomplete questionnaire submissions were excluded from analysis, and responses were screened for completeness before statistical analysis.”** |  |
| 12d. Statistical methods | Cross-sectional study—If applicable, describe analytical methods taking account of sampling strategy | Anonymized manuscript:  Pg 3 |
|  | **“… participants were recruited using convenience sampling, supplemented by snowball sampling to expand recruitment.”** |  |
| 12e. Statistical methods | Describe any sensitivity analyses | Anonymized manuscript:  n/a |
|  | **“Not applicable – no sensitivity analyses were required for this descriptive cross-sectional study.”** |  |
| **RESULTS** | | |
| 13a. Participants | Report the numbers of individuals at each stage of the study—e.g., numbers potentially eligible, examined for eligibility, confirmed eligible, included in the study, completing follow-up, and analysed. | Anonymized manuscript:  Pg 5 |
|  | “**… a total of 221 community pharmacists were included in the analysis, exceeding the minimum required sample size of 217.**” |  |
| 13b. Participants | Give reasons for non-participation at each stage. | Anonymized manuscript:  n/a |
|  | **“Not applicable – participation was voluntary and anonymous via online survey; reasons for non-participation were not collected.”** |  |
| 13c. Participants | Consider use of a flow diagram. | Anonymized manuscript:  n/a |
|  | **“Not applicable – the study used a descriptive cross-sectional design with convenience and snowball sampling; therefore, a participant flow diagram was not required.”** |  |
| 14a. Descriptive data | Give characteristics of study participants (e.g., demographic, clinical, social) and information on exposures and potential confounders. | Anonymized manuscript:  Pg 5–6, Table 1 |
|  | **“… participant characteristics including age, gender, educational level, pharmacist position, pharmacy type, and professional experience were summarized (Table 1).”** |  |
| 14b. Descriptive data | Indicate the number of participants with missing data for each variable of interest. | Anonymized manuscript:  Pg 3–4 |
|  | **“No missing data were reported, as only complete questionnaire responses were included in the analysis.”** |  |
| 14c. Descriptive data | Cohort study—Summarise follow-up time (e.g., average and total amount). | Anonymized manuscript:  n/a |
|  | **“Not applicable – follow-up time was not reported because this study employed a cross-sectional design, in which no longitudinal follow-up was conducted.”** |  |
| 15. Outcome data | Cross-sectional study—Report numbers of outcome events or summary measures. | Anonymized manuscript:  Pg 6–8, Tables 2–4 |
|  | **“… pharmacists demonstrated moderate knowledge, highly positive attitudes, and generally good but inconsistent practice behaviors in self-medication consultations (Tables 2–4).”** |  |
| 16a. Main results | Give unadjusted estimates and, if applicable, confounder-adjusted estimates and their precision (e.g., 95% confidence interval). Make clear which confounders were adjusted for and why they were included. | Anonymized manuscript:  Pg 8–9, Table 5 |
|  | **“… multiple linear regression identified knowledge and attitudes as significant predictors of practice scores after adjusting for demographic and professional characteristics (Table 5).”** |  |
| 16b. Main results | Report category boundaries when continuous variables were categorized. | Anonymized manuscript:  Pg 4 |
|  | **“… knowledge scores were categorized into good, moderate, and poor levels based on Bloom’s cut-off criteria.”** |  |
| 16c. Main results | If relevant, consider translating estimates of relative risk into absolute risk for a meaningful time period. | Anonymized manuscript:  n/a |
|  | **“Not applicable – this study was a cross-sectional KAP survey that did not involve any risk estimates or longitudinal outcome measures.”** |  |
| 17. Other analyses | Report other analyses done—e.g., analyses of subgroups and interactions, and sensitivity analyses. | Anonymized manuscript:  Pg 7–8, Tables 2–4; plus Appendix 1 |
|  | **“… additional analyses included item difficulty index (Pd) for knowledge items, relative importance index (RII) to rank attitude and practice items, and comparative analyses across demographic and professional characteristics (Tables 2–4; see also Appendix 1).”** |  |
| **DISCUSSION** | | |
| 18. Key results | Summarise key results with reference to study objectives. | Anonymized manuscript:  Pg 16 |
|  | **“… community pharmacists had moderate knowledge, highly positive attitudes, and generally good but inconsistent practices, with attitudes identified as the primary predictor of practice.”** |  |
| 19. Limitations | Discuss limitations of the study, taking into account sources of potential bias or imprecision. Discuss both direction and magnitude of any potential bias. | Anonymized manuscript:  Pg 19 |
|  | **“… the cross-sectional design limits causal inference, and the self-administered questionnaire may be subject to social desirability bias.”** |  |
| 20. Interpretation | Give a cautious overall interpretation of results considering objectives, limitations, multiplicity of analyses, results from similar studies, and other relevant evidence. | Anonymized manuscript:  Pg 16–18 |
|  | **“… findings suggest that pharmacists’ consultation practices are shaped by behavioral determinants within the COM-B framework, with attitudes playing a central role, highlighting the need for targeted interventions to strengthen capability and opportunity in community pharmacy practice.”** |  |
| 21. Generalisability | Discuss the generalisability (external validity) of the study results. | Anonymized manuscript:  Pg 19 |
|  | **“… the findings provide context-specific insights from community pharmacy practice in Indonesia, which may inform similar settings in low- and middle-income countries.”** |  |
| **OTHER INFORMATION** | | |
| 22. Funding | Give the source of funding and the role of the funders for the present study and, if applicable, for the original study on which the present article is based. | Title page:  Pg 2 |
|  | “**Funding … Republic of Indonesia … (Grant No. …). The funders had no role in the study design, data collection, analysis, interpretation, or manuscript preparation.**” |  |
